# Pain, lactate, and anesthetics: intertwined regulators of tumor metabolism and immunity

**DOI:** 10.3389/fonc.2025.1534300

**Published:** 2025-03-17

**Authors:** Qinghai Lan, Aiping Ouyang, Yijian Chen, Youchun Li, Baolin Zhong, Simin Deng

**Affiliations:** Department of Anesthesiology, Ganzhou People's Hospital, Ganzhou, Jiangxi, China

**Keywords:** anesthesia, lactate, pain, cancer progression, metabolism, immunity

## Abstract

Patients with advanced cancer frequently endure severe pain, which substantially diminishes their quality of life and can adversely impact survival. Analgesia, a critical modality for alleviating such pain, is now under scrutiny for its potential role in cancer progression, a relationship whose underlying mechanisms remain obscure. Emerging evidence suggests that lactate, once considered a metabolic byproduct, actively participates in the malignant progression of cancer by modulating both metabolic and immunological pathways within the tumor microenvironment. Furthermore, lactate is implicated in the modulation of cancer-related pain, exerting effects through direct and indirect mechanisms. This review synthesizes current understanding of lactate’s production, transport, and functional roles in tumor cells, encompassing the regulation of tumor metabolism, immunity, and progression. Additionally, we dissect the complex, bidirectional relationship between lactate and pain, and assess the impact of anesthetics on pain relief, lactate homeostasis, and tumorigenesis.

## 1 Introduction

Approximately 80% of patients with advanced cancer encounter cancer-related pain, resulting from tissue infiltration, compression, and destruction by tumor growth or metastasis (1), as well as from cancer treatments (2). This pain significantly impairs patients’ quality of life and can reduce survival rates (3). Thus, effective pain management is crucial. The World Health Organization advocates a multi-step analgesic ladder, including non-opioid and opioid analgesics, tailored to the pain’s severity (4, 5). Despite opioids’ central role in cancer pain management, their adverse effects, notably tolerance, diminish their efficacy. Consequently, alternative multimodal strategies, such as anesthesia, should be integrated into therapy (6). Anesthetics like lidocaine, ketamine, and gabapentinoids have demonstrated efficacy in managing chronic pain (7). Recent research implicates these agents in modulating cancer progression via effects on tumor immunity (8) and metabolism (9). Intriguingly, pain itself may influence tumorigenesis by altering biochemical processes, including lactic acid production in the tumor microenvironment.

Once regarded as merely a metabolic byproduct, lactate has now emerged as a multifunctional metabolite with crucial roles in cellular physiology: it serves as a critical energy substrate for mitochondrial respiration, a principal precursor for gluconeogenesis, and exerts signaling functions (10). In the context of oncology, the Warburg effect—a phenomenon where tumor tissues exhibit heightened glucose consumption compared to surrounding tissues—has been extensively studied. This metabolic reprogramming is characterized by the preferential routing of glucose through aerobic glycolysis, even in the presence of oxygen, resulting in the production of lactate at the expense of carbon dioxide. This metabolic shift results in elevated levels of lactate within both the intracellular and extracellular compartments of the tumor microenvironment (TME) (11). Furthermore, lactate shuttling within the TME facilitates a metabolic symbiosis among cancer cells dispersed throughout the tumor, allowing for the exchange of nutrients and energy-rich molecules (12). Under conditions of microenvironmental stress, histone lysine lactylation (Kla) accumulates at gene promoters, driving the production of lactate and modulating gene expression patterns that influence tumor growth and metastasis (13).

In summary, lactate emerges as a pivotal metabolite in tumor progression, with its intricate interplay with pain suggesting a broader clinical role for anesthesia in cancer therapy beyond pain relief. This review delves into the nexus of lactate and tumor biology, including the bidirectional dynamics between pain and lactate. We also assess the impact of anesthesia on pain management, lactate metabolism, and cancer progression. These insights underscore the potential for refined anesthetic strategies to enhance the efficacy of pain management and impede cancer progression, ultimately aiming to improve clinical outcomes and the quality of life for oncology patients.

## 2 The effect of lactate on tumors

### 2.1 Biological basis of lactate in cancer

Research has shown that hypoxic conditions strongly up - regulate the expression of Hypoxia - inducible factor - 1α (HIF - 1α). HIF - 1α, in turn, induces the expression of glucose transporters (GLUTs) and the monocarboxylate transporter 4 (MCT4). This induction redirects glucose (Glc) metabolism towards hypoxic glycolysis, bypassing the tricarboxylic acid (TCA) cycle. Instead, pyruvate (Pyr), the primary product of glycolysis, is shunted towards conversion into lactate by lactate dehydrogenase (LDH), augmenting the rate of energy production even under oxygen-limited conditions (14). In addition to insufficient oxygen supply and demand leading to lactic acid production in the body, insufficient organ perfusion (15) actually promotes increased lactic acid production and lactic acid accumulation as well. Furthermore, nutritional status also has a regulatory role in lactate. In a study by Wang et al, it was found that the increase in lactate in obese mice mainly originated from white adipocytes, and the underlying mechanism was related to the increased expression of LDHA in adipocytes (16) In a seminal hypothesis proposed by Otto Warburg in 1956, it was posited that tumor cells, even in the presence of oxygen, favor glycolysis over oxidative phosphorylation (OXPHOS) for energy generation. This concept has become the cornerstone of cancer metabolism research (17). Subsequent investigations have revealed a paradoxical metabolic landscape within tumors, with cells distant from blood vessels exhibiting a heightened propensity for aerobic glycolysis compared to those in proximity to vascular structures. The accelerated proliferation of tumor cells outstrips angiogenic processes, rendering aerobic glycolysis a predominant energy source and culminating in the accumulation of lactic acid within the tumor microenvironment (TME). The conventional view is that the reason for this phenomenon may be mitochondrial functional impairment and activation of glycolytic genes by oncogenic signals (e.g., HIF-1, Myc), but Hyllana et al. (18) in 2022 proposed a new idea: the capacity saturation of the mitochondrial NADH shuttle system (including the Malate-Aspartate Shuttle and the Glycerol-3- Phosphate Shuttle) capacity saturation is the key reason for triggering aerobic lactic acid fermentation by the specific mechanism of NADH accumulation in the cytoplasm when the rate of glycolysis exceeds the transport capacity of the NADH shuttle system. As a result of NADH accumulation, the NAD^+^/NADH ratio decreases, inhibiting the activity of GAPDH, a key enzyme in glycolysis. To sustain glycolysis (and ensure ATP supply), the cell reduces pyruvate to lactate via LDH and regenerates NAD^+^. Beyond aerobic glycolysis, lactate in the TME also originates from the catabolism of glutamine (Gln) (19–21). Gln serves a dual role, supplying a carbon scaffold for lactate synthesis and generating NH4+ to counterbalance the acidosis resulting from lactate accumulation, thereby creating a protective niche for tumor cells within the TME (22).

Elucidating the transport mechanisms of lactate, it becomes evident that lactate is shuttled between intracellular and extracellular compartments primarily by monocarboxylic acid transporter proteins (MCT1-4) and sodium-dependent transporter proteins (SMCT1-2). The vectorial movement of lactate is dictated by the concentration gradient across the cellular membranes (23). Beyond its role as a metabolic byproduct of aerobic glycolysis in tumor cells, lactate has emerged as a pivotal signaling molecule, orchestrating the progression of tumor cells. Contemporary research has unveiled that lactate is capable of engaging specific lactate receptors, G-protein-coupled receptor 81 (GPR81, also known as HCAR1) and GPR132 (G2A), thereby modulating downstream signaling cascades that influence tumor cell behavior. Notably, GPR81, which is frequently overexpressed in cancer patients, serves as a critical lactate receptor, through which lactate is implicated in the modulation of tumor metabolism, progression, and immune interactions (24, 25).

Lactate is cleared in a total of two ways. The first involves the oxidation of lactate to form pyruvate and subsequently acetyl coenzyme A, which is then used in the tricarboxylic acid cycle to form CO2, water and provide energy. The other is the gluconeogenesis of lactate to glucose in the liver and skeletal muscle cells in response to hormones such as glucagon and cortisol. Under normal conditions, the liver is the primary site in the body that exhibits the highest lactate clearance. When liver function is impaired, lactate clearance decreases, leading to lactate accumulation (26).

### 2.2 The role of lactate in tumor metabolism

**Figure 1:**
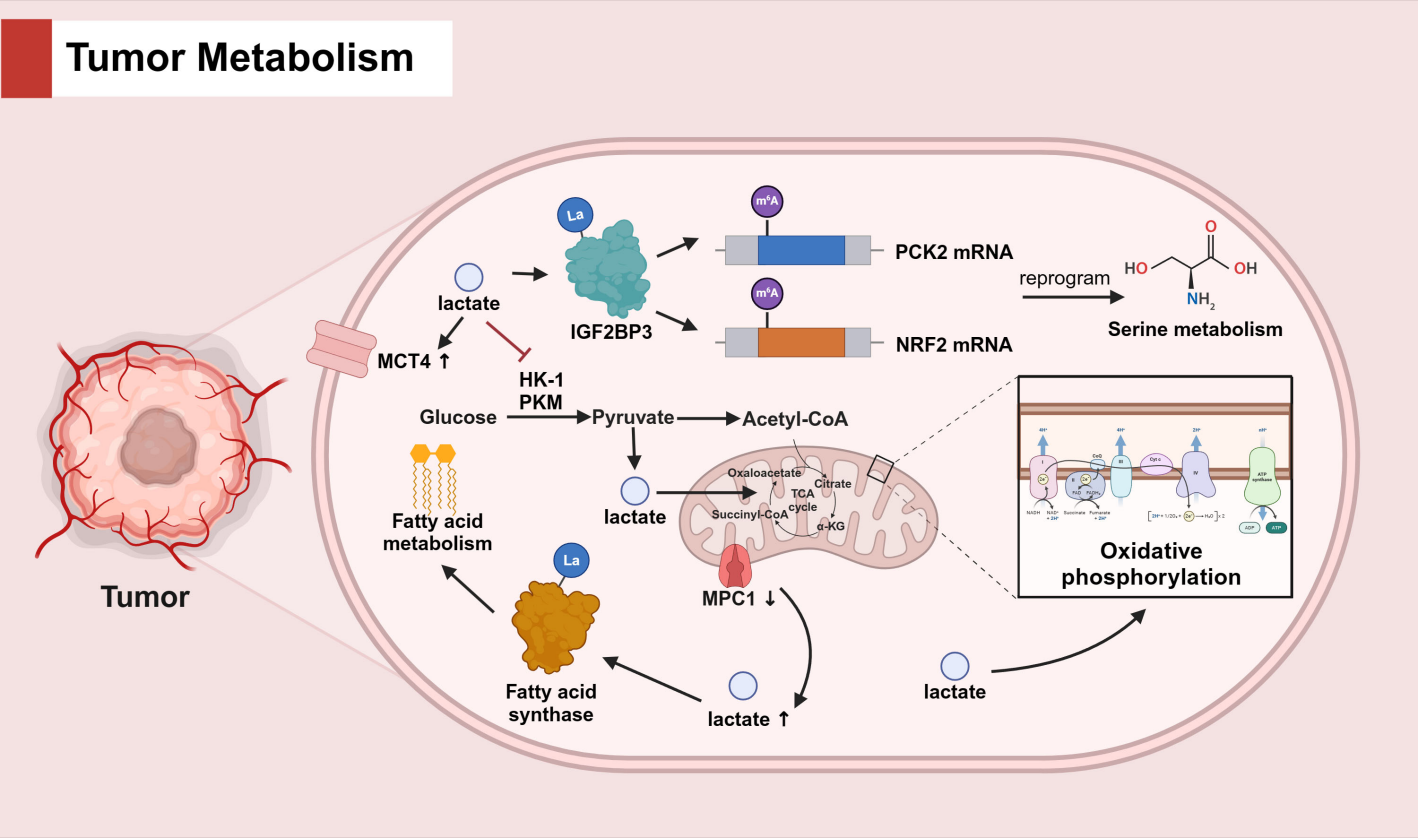
The role of lactate in tumor metabolism.

Histone lysine lactylation (Kla), an epigenetic modification once overlooked, has emerged as a critical regulator in the complex orchestration of cancer progression, exerting its influence over tumor metabolism, immunity, and additional biological mechanisms (27). Proteomic dissection of hepatocellular carcinoma (HCC) has unveiled the pervasive impact of Kla on a spectrum of bio-metabolic pathways, encompassing carbohydrate, amino acid, fatty acid, and nucleotide metabolism (28). The modus operandi of Kla is presumed to be its predominant interaction with key enzymes that govern these metabolic routes (29). In a seminal study, Zhang et al. pinpointed 27 lactylation-related genes (LRGs) in osteosarcoma (OS) that were intimately linked to amino acid metabolism, as elucidated by KEGG and GO annotations (30). In the tumor microenvironment (TME), lactate accumulation and its subsequent lactylation of IGF2BP3 were discovered to upregulate the expression of PCK2 and NRF2, thereby instigating serine metabolic reprogramming, augmenting the availability of S-adenosylmethionine (SAM), and conferring drug resistance in hepatocellular carcinoma (31). In the context of uveal melanoma (UM), lactate accumulation was observed to amplify the expression of transporter proteins and intensify OXPHOS activity (32). Moreover, lactate has been recognized to suppress the expression of glycolytic enzymes, such as HK-1 and PKM, while simultaneously promoting the expression of TCA cycle enzymes, including SDHA and IDH3G, thereby curbing cellular glycolysis and preserving mitochondrial homeostasis in non-small cell lung cancer (33). Delving into the interplay between lactate and fatty acid metabolism, Gao et al. demonstrated that the modulation of mitochondrial pyruvate carrier 1 (MPC1) could orchestrate lactate levels, influencing the lactylation status of fatty acid synthase K673 and, consequently, inhibiting fatty acid synthase activity (34). (Refer to **Figure 1** for the detailed mechanisms about the role of lactate in tumor metabolism).

In tumor cells, lactate accumulation induces IGF2BP3 lactylation and enhances m6A methylation of PCK2 and NRF2 mRNAs, reprogramming serine metabolism. Lactate also upregulates MCT4 expression to promote lactate transport and increase OXPHOS activity. Lactate affects glucose metabolism by inhibiting cellular glycolysis and maintaining mitochondrial homeostasis through the inhibition of glycolytic enzymes (HK-1, PKM) and the promotion of TCA cycle enzymes. Furthermore, MPC1 knockdown causes lactate accumulation, promotes fatty acid synthase lactylation and affects fatty acid metabolism.

### 2.3 The role of lactate on tumor immunity

Lactic acid is increasingly acknowledged for its role in promoting tumor progression, through inducing tumor acidosis and suppressing antitumor immunity. The TME is mainly composed of tumor cells, immune cells and supporting cells (e.g. fibroblasts, stromal cells and endothelial cells), as well as biologically active molecules (35). On the one hand, lactic acid interacts with immune cells and interferes with their proliferation, differentiation and immune function. On the other hand, lactate affects basal cells and endothelial cells and promotes tumor deterioration phenotypes such as basement membrane (BM) remodeling, epithelial-mesenchymal transition (EMT), metabolic reprogramming, angiogenesis and drug resistance.

Shang et al. devised a sophisticated risk prediction model revealing a positive correlation between lactate levels and the presence of CD4 T cells, CD8 T cells, M1 macrophages, and the activation of multiple immune pathways (36). In a parallel development, Yang et al. established a lactylation-associated model, highlighting the strong correlation between elevated lactylation and immune cell infiltration, particularly macrophages, alongside genetic instability. Notably, this model suggests that highly lactylated tumors are substantially more prone to immune evasion (37). Current research is intently focused on how lactic acid orchestrates tumor immunity by modulating T cell proliferation, differentiation, and function. The accrual of lactate leads to the inhibition of glyceraldehyde-3-phosphate dehydrogenase (GAPDH) and phosphoglycerate dehydrogenase (PGDH), key executors of NAD-dependent enzymatic reactions, thereby triggering reductive stress. This sequence of events results in a decline of serine, an essential metabolite, as lactate suppresses the expression of GAPDH and PGDH in T cells, thereby curtailing T cell proliferation (38). The chemokine IL-8 is known to enhance the infiltration of regulatory T-cells (Tregs) through the DAPK1/pyruvate kinase/lactate axis in response to fluctuations in tumor cell density (39). Tregs, adept at lactate uptake, were shown by Rao et al. to increase the lactylation of MOESIN and its subsequent binding to TGFβ in a pH-dependent manner, promoting the differentiation of conventional CD4+ T cells into Tregs (40). Lactate also plays a pivotal role in the differentiation of CD8+ T cells. Wenes et al. demonstrated that the inhibition of the mitochondrial pyruvate carrier (MPC) enhances the production of acetyl coenzyme-A, which in turn increases histone acetylation and chromatin accessibility of pro-memory genes, propelling CD8+ T cells towards a memory phenotype (41). In the context of tumor-associated macrophages (TAMs), high concentrations of lactic acid activate the MCT1/NF-kB/COX-2 pathway, inducing high levels of PD-L1 in neutrophils and thereby inhibiting the antitumor functions of T cells (42).

Lactate’s influence on macrophage-associated tumor immunity extends to both direct activation and indirect effects on macrophage recruitment and polarization. Shi et al. discovered that APQ9, a transporter protein vital for water and glycerol transport in macrophages, is less responsive to lactate stimulation in APQ9 knockout macrophages, which also exhibit significantly reduced lactate transport (43). This finding implies a role for APQ9 in mediating lactate transport within macrophages. Furthermore, GPR65, identified as a lactate sensor in TAMs, detects lactate in the TME and initiates the downstream cAMP/PKA/CREB signaling pathway, promoting the release of high-mobility group box 1 (HMGB1) from TAMs and thereby accelerating tumor progression (44). Lactate has also been shown to stimulate the release of interleukin (IL)-1β from TAMs in an inflammatory vesicle-dependent manner, with IL-1β further promoting TAM recruitment through the induction of C-C motif chemokine ligand 2 from tumor cells (45). In the TME, TAMs shift towards an M2 phenotype in response to lactate stimulation, thereby promoting tumor progression. Interestingly, despite exhibiting an M2 phenotype, TAMs display a metabolic profile akin to M1 macrophages, characterized by high glycolytic activity (46). M0 macrophages, upon lactate uptake from the TME, undergo H3K18 lactylation and M2 polarization. In pituitary adenomas (PA), lactate produced by PA cells induces M2 polarization of TAMs and stimulates the secretion of CCL17 by TAMs, thereby promoting PA cell invasion through the mTORC2 and ERK signaling pathways (47). In a striking demonstration of metabolic plasticity, D-lactate was found to induce the phenotypic switch of M2-type TAMs to M1-type TAMs by modulating the PI3K/Akt pathway (48). These findings collectively suggest a dynamic, bidirectional regulatory relationship between TAMs and lactate.

In the case of B cells, lactic acid may affect their metabolic changes and modulate B cell immune functions. Senescent B cells are hypermetabolic and are more likely to shift from oxidative phosphorylation to anaerobic glycolysis, leading to increased lactate secretion. Lactate induces autoimmune pathogenic B cells and stimulates autoimmune antibody secretion (49). LDHA is highly expressed in various tumors, and Feng et al. found that LDHA is highly expressed in diffuse large B-cell lymphoma (DLBC). Further studies revealed that LDHA regulates the metabolism, proliferation and invasion of Raji cells through feline sarcoma-related protein (FER), which may be a potential therapeutic target (50). B-cell lymphomas predominantly use MCT-1 to export lactate, Ernesto et al. found that inhibition of MCT-1 promoted the anti-tumour function of CAR T-cell therapy, and that this combination therapy was effective in improving cytotoxicity *in vitro* and tumor clearance *in vivo (* 51).

NK cells are able to synthesize a variety of killing mediators such as IFN γ, which directly exerts tumor clearance function. Lactic acid reduces IFN γ production by inhibiting nuclear factor of activated T cells (NFAT) in NK cells (52). In addition, lactic acidosis caused by the SIX1/LDHA axis contributes to NK cell dysfunction in pancreatic cancer (53). Lactate also inhibits lipid biosynthesis and antitumor activity of NK cells by decreasing the expression of peroxisome proliferator-activated receptor γ (PPARγ) (54).

DCs, as antigen-presenting cells, are able to activate the immune response by processing and presenting antigens via MHC-II and MHC-I. Li et al. concluded that accumulation of lactate in tumors limits the ability of DCs to recognize and present antigens. In addition, lactate leads to inhibition of DC differentiation and promotes the production of the immunosuppressive cytokine IL-10 by DCs and inhibits the secretion of the pro-inflammatory factor IL-12 (55).

In TME, cancer-associated fibroblasts (CAFs) promote basement membrane remodeling (BMR) mainly through the secretion of type I collagen, as well as EMT, which in turn facilitates tumor invasion. Lactate enhances NUSAP1 nuclear translocation, recruiting the JUNB-FRA1-FRA2 transcriptional complex to activate DESMIN expression in CAFs. This promotes M2 macrophage polarization via IL-8 secretion, supporting tumor-associated macrophage (TAM) recruitment (56). In addition, Apicella et al. found that the use of tyrosine kinase inhibitors (TKIs) induced metabolic changes in tumor cells leading to an increase in lactate production, which contributed to the overproduction of HGF by CAF, a process involved in tumor drug resistance (57). Chen et al. concluded that lactate mediates the release of IL-8 from endothelial cells after entering endothelial cells via MCT1, as well as the migration of endothelial cells, which promotes angiogenesis (58).

**Figure 2:**
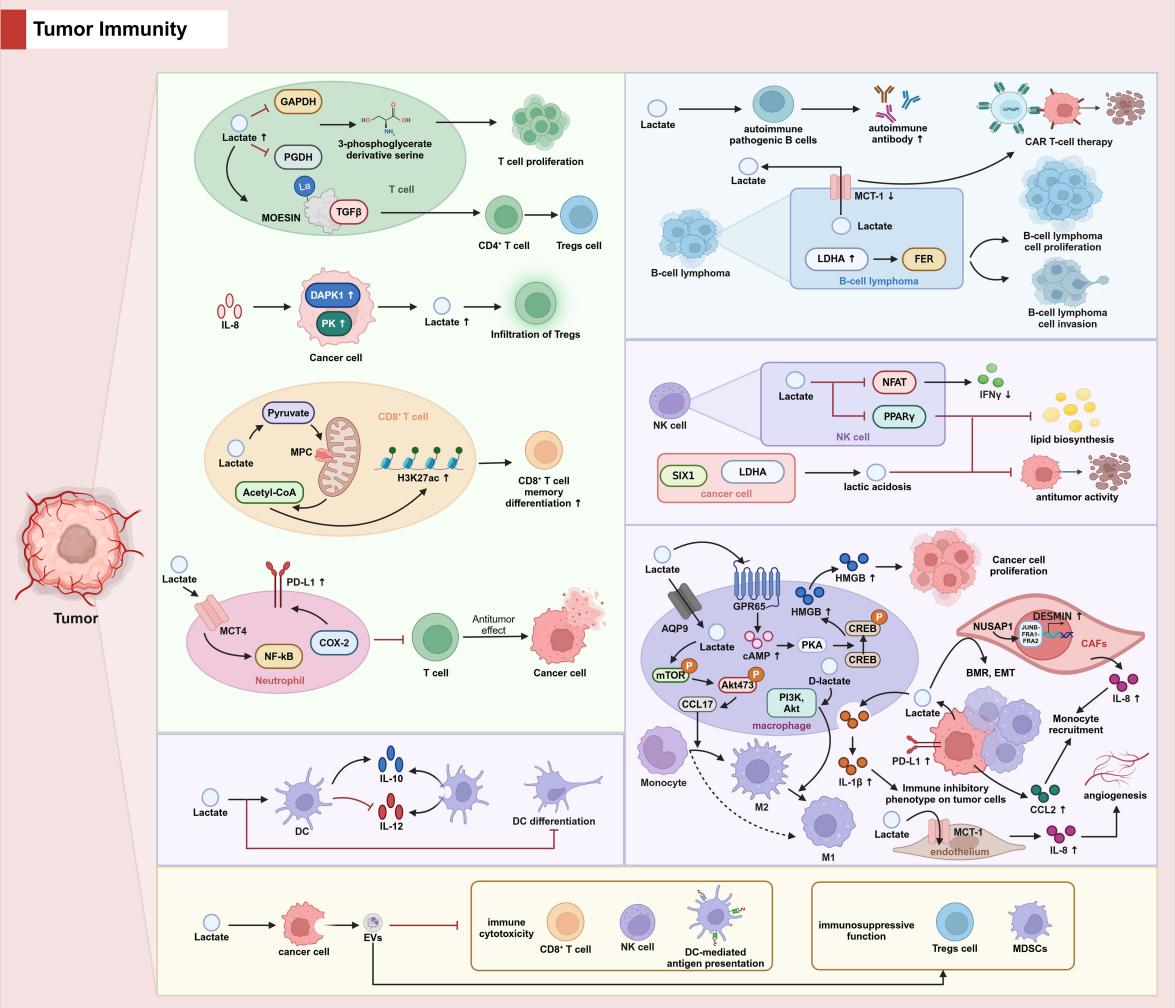
The role of lactate in tumor immunity.

In addition to mediating tumor immunity by regulating immune cell proliferation, differentiation and the exercise of immune functions through diverse pathways, it has been proposed that lactic acid mediates the release of extracellular vesicles (EVs) from cancer cells, and that tumor-derived EVs inhibit various types of immune cytotoxicity, such as CD8 T-cells and NK-cells, as well as DC-mediated antigen presentation and enhance the immunosuppressive function of Tregs and MDSCs, thus promoting the immune escape of cancer cells (59). (The effect of lactic acid on tumor immunity is shown in **Figure 2**, and differences in the mechanisms by which lactate regulates immune cell function in different tumors are shown in **Table 1**).

**Table 1:** Mechanisms of lactate regulation of immune cell function in different tumor types.

Cancer type	Functions on immune cells	Ref.
Gastric cancer	curtailing T cell proliferation	(38)
Hepatocellular carcinoma	promoting the differentiation of conventional CD4+ T cells into Tregs	(40)
Hepatocellular carcinoma	inducing high levels of PD-L1 in neutrophils and thereby inhibiting the antitumor functions of T cells	(42)
Hepatocellular carcinoma	induce the phenotypic switch of M2-type TAMs to M1-type TAMs	(48)
Hepatocellular carcinoma, melanoma	inhibits lipid biosynthesis and antitumor activity of NK cells	(54)
Melanoma, leukemia	propelling CD8+ T cells towards a memory phenotype	(41)
Melanoma	inhibiting nuclear factor of activated T cells (NFAT) in NK cells	(52)
Colon cancer	stimulate an M2-like polarization	(43)
Glioma	induces HMGB1 release from TAM	(44)
Ovarian cancer, lung cancer, glioma	promoting TAM recruitment	(45)
Lung cancer	activating DESMIN transcription in cancer-associated fibroblasts (CAFs), which in turn foster M2 polarization by secreting IL-8, thereby recruiting TAMs or promoting macrophage activity	(56)
Pituitary adenoma	induces M2 polarization of TAMs	(47)
B-cell lymphoma	regulates the metabolism, proliferation and invasion of Raji cells	(60)
B-cell lymphomas	regulates the metabolism, proliferation and invasion of Raji cells	(51)
Pancreatic cancer	contributes to NK cell dysfunction	(53)

In TME, lactate inhibits 3-phosphoglycerate derived serine by suppressing the expression of GADPH and PGDH in T cells, leading to inhibition of T cell proliferation. In addition, lactate promotes the differentiation of CD4^+^ T cells to Tregs cells by promoting MOESIN lactylation and MOESIN binding to TGFβ. IL-8 upregulates DAPK1 and PK expression in tumor cells, promotes lactate secretion by tumor cells and causes Treg cell infiltration. In CD8+ cells, MPC maintains lactate oxidation to sustain cytotoxic T cell antitumor functions. In neutrophils, lactate enters neutrophils via MCT and promotes PD-L1 expression via the NF-κB/cox pathway and inhibits tumor killing function of T cells. In macrophages, lactate activates GPR65, promotes downstream cAMP/PKA/CREB activation, facilitates HMGB1 secretion and promotes tumor cell proliferation. Lactate also enters macrophages via AQP9, promotes the release of CCL17 via the mTOR/ERK pathway, and promotes macrophage polarization towards M2. D-lactate, on the other hand, promotes macrophage conversion from M2 to M1 via the PI3K/Akt pathway. Lactic acid secreted by tumor cells into macrophages promotes the secretion of IL-1β from even cells, and IL-1β induces an immunosuppressive phenotype in tumor cells. Furthermore, CCL2 secreted by tumor cells as well as IL-8 secreted by CAFs induces macrophage recruitment. Lactate induces autoimmune pathogenic B cells and stimulates the secretion of autoimmune antibodies. LDHA is highly expressed in diffuse large B-cell lymphoma (DLBC) and regulates the metabolism, proliferation and invasion of Raji cells via FER. Inhibition of MCT-1 in B-cell lymphoma promotes the anti-tumor function of CAR T-cell therapy. Lactate inhibition of NFAT in NK cells reduces the production of the killing factor IFN γ. Lactate also inhibits lipid biosynthesis and anti-tumor activity in NK cells by down-regulating PPARγ expression. Lactic acidosis induced by the SIX1/LDHA axis in cancer cells also leads to NK cell dysfunction. Lactate inhibits the differentiation of DC cells, promotes the production of the immunosuppressive cytokine IL-10 by DC cells, and inhibits the secretion of the pro-inflammatory factor IL-12. Lactic acid mediates the release of EV from cancer cells, which in turn inhibits various immune cytotoxicity such as CD8 T cells and NK cells as well as DC-mediated antigen presentation, and enhances the immunosuppressive function of Tregs and MDSCs, thereby promoting the immune escape of cancer cells.

### 2.4 The role of lactate on tumor progression

Lactate, a pivotal energy substrate for cancer cells, is frequently correlated with advanced tumor progression, encompassing cancer cell proliferation, invasion, and metastasis. Compelling studies demonstrate that the intravenous administration of Veillonella parvula, an anaerobic bacterium capable of fermenting lactic acid, substantially diminishes lactate levels and curbs tumor cell proliferation and metastasis (61). In the context of breast cancer, lactate within the TME triggers the assembly of the hydroxycarboxylic acid receptor 1 (HCAR1), thereby activating downstream RAS and PI3K oncogenic signaling pathways, which are instrumental in promoting cancer progression (62). The accumulation of lactate in breast cancer is also linked to heightened heterogeneous gene expression and the histomorphogenesis associated with invasion (63). Recent discoveries highlight the correlation between a low pH environment, a hallmark of TME acidosis due to lactate accumulation, and the elevated invasive capacity of tumor cells. Carbonic anhydrase IX (CA9), a biomarker of tumor invasion, exerts a significant regulatory role in modulating this acidic TME (64). The lactate transporter protein MCT4, in conjunction with CD147, is implicated in the matrix metalloproteinase 14 (MMP14)-mediated degradation of the extracellular matrix (ECM), thereby facilitating breast cancer cell invasion (65). Conversely, lactate oxidase (LOX) downregulates lactate levels by impeding MCT1 and MCT4, thereby disrupting the lactate-driven oncogenic pathway (62). The suppression of GPR81 in breast cancer cells has been shown to inhibit cancer cell migration and invasion, potentially due to the impairment of glycolysis and lactate-dependent ATP production in cancer cells (66).

Beyond the direct tumor-promoting effects of lactic acid, pivotal components within the lactate production cascade, specifically within the context of aerobic glycolysis, are deeply implicated in oncogenic processes. Lactate dehydrogenase A (LDHA), a crucial enzyme in the terminal phase of glycolysis, exhibits heightened expression in cancerous tissues. Hou et al. have delineated that the upregulation of LDHA expression augments lactic acid production and histone lysine lactylation, diminishes tumor cell adhesion, and ultimately fuels the proliferation, invasion, and migration of breast cancer cells (67). Chen et al. have uncovered that ZDHHC9-mediated palmitoylation of LDHA enhances lactic acid production, thereby promoting the proliferation and growth of pancreatic tumor cells (68). The efficacy of LDH inhibitors in curbing breast cancer cell proliferation, motility, and invasion reiterates the indispensable role of lactate in cancer progression (69). Moreover, the overexpression of paired homology structural domain transcription factor 2 (PITX2) in ovarian cancer cells has been observed to induce the nuclear accumulation of LDHA, resulting in elevated lactate concentrations. The silencing of LDHA attenuates lactate production and subsequently inhibits the proliferative rate of tumor cells (70). The initial rate-limiting enzymes of glycolysis, hexokinase 1 (HK1) and hexokinase 2 (HK2), are markedly overexpressed in breast cancer, with the suppression of HK expression leading to the dampening of breast cancer cell proliferation, invasion, and migration (71). In gastric cancer, SALL4 has been shown to stimulate the proliferation, invasion, and migration of gastric cancer cells through the upregulation of HK-2 (72). HK-2, a key glycolytic enzyme, also plays a regulatory role in the pyroptosis of cancer cells induced by tretinoin (TPL) (73).

The activation of glycolysis in non-malignant cells within the tumor microenvironment (TCM) also plays a crucial role in fostering the neoplastic development. Once activated, cancer-associated fibroblasts (CAFs) exhibit a heightened rate of aerobic glycolysis, which, when co-cultured with nasopharyngeal carcinoma (NPC) cells, significantly amplifies the migratory capacity of these cancer cells (74). Furthermore, the glycolytic activation of bone marrow stromal cells (BMSCs) induced by exosomes derived from lung cancer A549 cells is instrumental in sculpting a pre-metastatic niche. This interaction promotes the proliferation and metastasis of A549 cells through a metabolic phenomenon known as the reverse Warburg effect, wherein the BMSCs engage in aerobic glycolysis to support the metabolic demands of the cancer cells (75).

**Figure 3:**
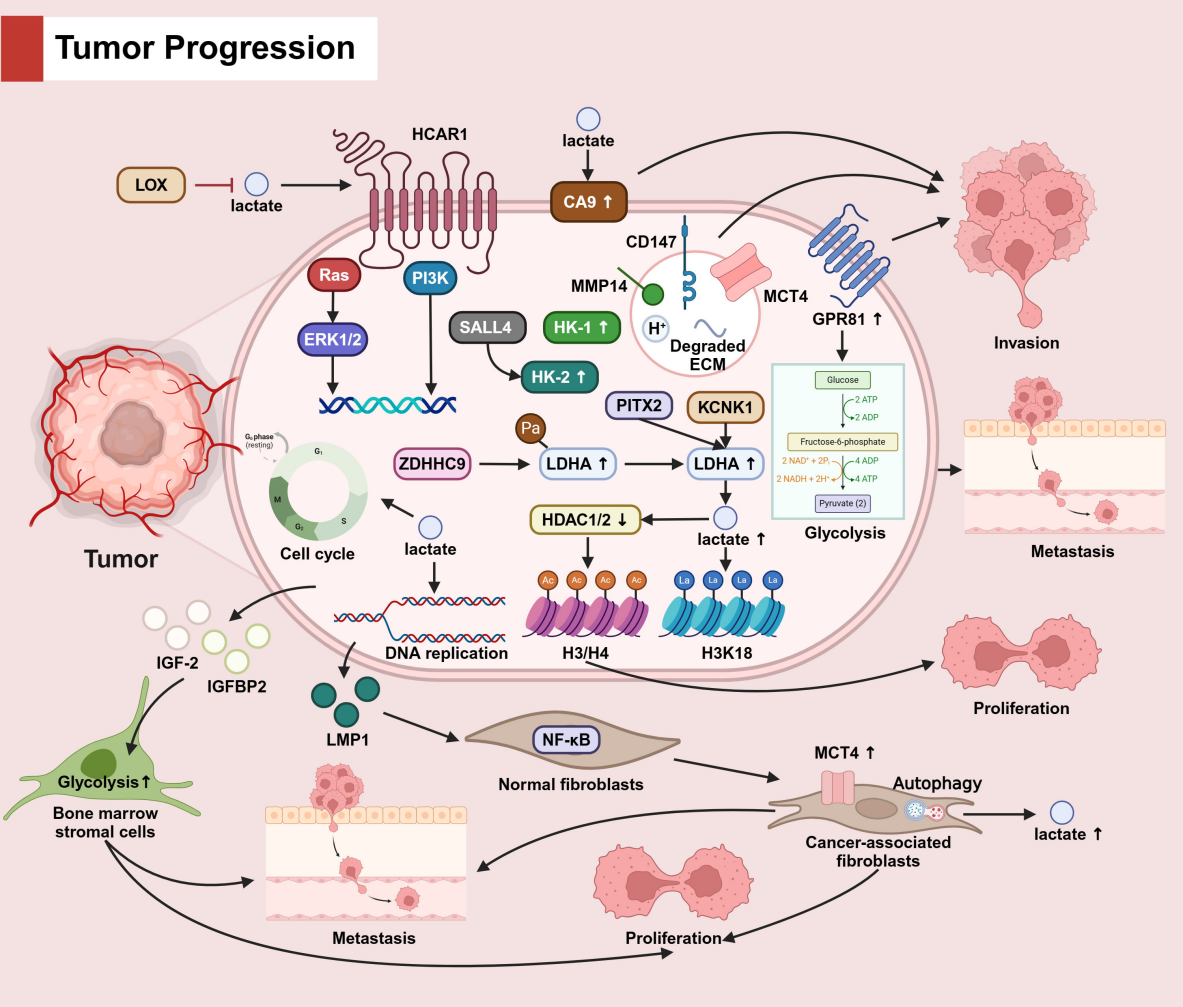
The role of lactate in tumor progression.

In tumor cells, lactate promotes tumor cell invasion by up-regulating CA9 expression. MCT4, along with CD147, MMP14, H+ and degraded ECM, promote tumour cell invasion via vesicular transport, and high expression of GPR8 fosters glycolysis, which also contributes to tumor invasion. In TME, LOX reduces lactate accumulation. Lactate activates TCAR1, which enhances cancer progression through downstream Ras and PI3K pathways. In addition, lactate facilitates tumor cell proliferation, invasion, and migration through the cell cycle and DNA replication. overexpression of HK-1 and HK-2 likewise has pro-cancer effects. PITX2 and KCNK1 as well as ZDHHC9-mediated palmitoylation up-regulate LDHA expression, promote lactate production, then lactylate H3K18 and down-regulate HDAC1/2 to inhibit H3/H4 acetylation, ultimately inhibiting tumor cell proliferation. LMP1 secreted by tumor cells induces the activation of normal fibroblasts into tumor-associated fibroblasts through the NF-κB pathway, promotes the high expression of MCT4 as well as the occurrence of autophagy, and ultimately fosters the proliferation and migration of tumor cells. IGF-2 and IGFBP2 secreted by tumor cells upregulate the glycolytic pathway of bone marrow stromal cells and promote tumor cell proliferation and metastasis. (The mechanism by which lactic acid affects tumor progression is seen in **Figure 3**).

## 3 The effect of lactate on cancer related pain

### 3.1 Biological basis of caner related-pain

When the human body is exposed to thermal, mechanical, or chemical stimuli exceeding a critical threshold, nociceptive neurons are activated, transmitting noxious signals from the periphery to the spinal cord. This neural transmission is instrumental in communicating the precise location and magnitude of the painful stimulus, as well as the subjective experience of pain and the modulation of the spinal cord’s descending feedback system (76). Pain can be broadly categorized into two forms: acute and chronic. Patients afflicted with cancer predominantly endure chronic pain, which emanates not only from the tumor’s direct compression and invasion but also from iatrogenic nerve damage and secondary inflammation induced by therapeutic interventions, such as chemotherapy and radiotherapy. Contemporary research implicates peripheral and central sensitization as the key mechanisms underlying chronic pain (77). Peripheral sensitization occurs when the inflammatory milieu, replete with agents like bradykinin, prostaglandins, H+, ATP, nerve growth factor (NGF), pro-inflammatory cytokines, and chemokines, activates receptors on nociceptors. These receptors include G protein-coupled receptors (GPCRs), transient receptor potential (TRP) channels, acid-sensitive ion channels (ASICs), two-pore potassium channels (K2P), and receptor tyrosine kinases (RTKs), thereby initiating pain signaling and perception (78). The paradigm of central sensitization encompasses three principal mechanisms: glutamate/NMDA receptor-mediated sensitization, disinhibition, and microglial activation (76). The glutamate/NMDA receptor-mediated pathway is activated by sustained noxious stimuli, which trigger C and Aδ fibers, leading to the release of various neurotransmitters. These neurotransmitters activate postsynaptic NMDA receptors, causing an influx of intracellular calcium and the subsequent activation of downstream signaling cascades (76). Disinhibition refers to the suppression of inhibitory interneurons, which normally tamp down the excitability of layer I output neurons by maintaining the release of gamma-aminobutyric acid (GABA) and/or glycine (Gly). This process can also involve the engagement of non-invasive Aβ primary afferent nerves in pain transmission due to the neuronal expression of excitatory PKCγ, culminating in allodynia. Microglial activation, induced by ATP and chemokines released from damaged peripheral nerves, results in central sensitization through the secretion of brain-derived neurotrophic factor (BDNF) and pro-inflammatory cytokines (76).

### 3.2 Regulation mechanisms of lactate on pain

A substantial body of research has established a robust correlation between lactic acid and pain perception. Micro-dialysis studies have demonstrated that both interstitial muscle and plasma lactate and glutamate concentrations are markedly elevated in individuals suffering from chronic widespread pain (CWP) as compared to asymptomatic subjects (79). Notably, elevated levels of lactic acid have been detected in the serum of patients grappling with cancer-related pain (80). The proposed mechanism involves the accumulation of lactic acid at postoperative incision sites, which, through a localized decrease in pH, may activate injury receptors and precipitate pain (81). Furthermore, in patients with bone cancer, an upregulation of ASIC1 has been observed, potentially contributing to heightened nociceptive sensitivity (82). Emerging research, however, suggests that lactic acid alone is not sufficient to elicit pain; rather, a combination of lactic acid, ATP, and H+, when administered intramuscularly, induces both fatigue and pain (83). The reason for this discrepant result may be that the study explored the tissue type of human thumb short adductor muscle. ASIC, a key receptor for lactic acid-triggered pain, is expressed in different tissues with different types (ASIC3 is expressed in the injurious neurons innervating the muscle (~50%) more than in the skin (~10%)). In addition, the thumb abductor digitorum is highly vascularized and highly innervated, so it is likely that the sensory activation profile of at least some other skeletal muscles will be different from that reported here. In addition, this study found that the metabolites administered alone were ineffective at low concentrations and only induced responses when administered at very high concentrations corresponding to vascular and muscle damage, suggesting that differences in setting threshold concentrations for metabolite-induced pain may be due to differences between human and animal models, leading to different conclusions.

Lactic acid’s role in chronic pain extends beyond direct stimulation of injury receptors, as it may also activate associated neurons. The inhibition of MCT4 expression in the dorsal root ganglion (DRG) of sensory neurons has been shown to downregulate the expression of pERK1/2 in the DRG (84), indicating that the transport of lactic acid, a byproduct of tumor cell glycolysis, may contribute to neuronal excitation and the manifestation of cancer-related pain. Astrocytes, known regulators of chronic pain, release lactate through glycolysis, facilitating an energy transfer to neurons via MCT, thereby activating the ERK/CREB signaling cascade and Fos gene expression, which are integral to the modulation of chronic pain (85). The inhibition of this lactate shuttle pathway has been proven effective in alleviating chronic pain (86). Additionally, lactate accumulation within the synaptic microenvironment upregulates the expression of microglial thioredoxin-interacting protein (TXNIP) mRNA, triggering downstream neuronal death, impacting synaptic plasticity, and perpetuating central sensitization (87).

### 3.3 Regulation mechanisms of pain on lactate

Investigations have revealed that pain serves a dual role, manifesting not only as an outcome of discomfort from noxious stimuli but also as a potent stressor that activates the hypothalamic-pituitary-adrenal-thyroid-gonadal (HPATG) axis, thereby influencing the endocrine system (88). Upon the genesis of pain in the brain, the hypothalamus is stimulated to secrete corticotropin-releasing hormone (CRH), gonadotropin-releasing hormone (GRH), and thyroid-releasing hormone (TRH), which in turn induce the secretion of hormones such as cortisol, dehydroepiandrosterone (DHEA), testosterone, progesterone, estrogen, triiodothyronine (T3), and thyroxine (T4). These hormones mediate pain control through immune, inflammatory, and glucoregulatory mechanisms. Studies in the toad species Bufo gargarizans have demonstrated that cortisol can decrease serum lactate levels, potentially through the enhancement of gluconeogenesis (89). However, similar research in humans is sparse. Plasma metabolomic analyses in men with hypogonadism have unveiled significant shifts in biochemical pathways, including the conversion of lactate to ketone bodies. Testosterone administration, conversely, has been shown to increase lactate levels and enhance glycolysis (90). Progesterone also plays a role in lactate regulation; Tomoka et al. observed changes in blood glucose, lactate, and insulin concentrations post-exercise in women across the early follicular, late follicular, and luteal phases, noting significantly higher lactate concentrations during the late follicular and luteal phases compared to the early follicular phase (91). This suggests a regulatory role for progesterone in lactate homeostasis. In contrast, Dragutinovic et al. reported reduced serum lactate accumulation during the mid-luteal phase in their examination of the menstrual cycle’s effect on exercise in women (92). These discrepancies may arise from variations in exercise intensity and lactate testing timing, with the precise mechanisms awaiting further elucidation. Estrogen, known to modulate glycolysis in cancer cells, has been further implicated by Zamer et al., who found that the co-inhibition of pyruvate kinase M2 (PKM2) expression with estrogen significantly reduced lactate levels and induced apoptosis in colorectal cancer cells more effectively than estrogen treatment alone (93). Marwali et al.’s study proposed that exogenous thyroid supplementation does not influence lactate levels in extracorporeal circulation and reperfusion (94). Despite thyroid hormones’ role in accelerating glucose metabolism, research on their impact on lactate is limited.

The feedback mechanisms of pain on lactate have been minimally explored, primarily due to a focus on symptomatic treatment mechanisms. Nonetheless, as a key signaling molecule, lactate may be intricately linked to various physiopathological processes, and the interplay between pain and lactate could have broader implications for downstream pathophysiological outcomes, warranting further investigation into this regulatory feedback loop.

## 4 Regulation of anesthesia

### 4.1 Mechanisms of pain modulation by anesthesia

Anesthetics are categorized into two fundamental types: general and local. General anesthetics induce a reversible state of unconsciousness, analgesia, and muscle relaxation, whereas local anesthetics are confined to producing a reversible loss of sensation in specific regions of the body without impairing consciousness. The realm of general anesthetics encompasses both inhalational and intravenous agents. Prominent among the inhalational anesthetics are ether, halothane, enflurane, and nitrous oxide. Intravenous anesthetics commonly utilized include propofol, thiopentone or thiopental sodium, and ketamine (95). Propofol exerts its effect by diminishing the opening duration of sodium channels, thereby blocking nerve conduction. In contrast, thiopentone or thiopental sodium inhibits nerve conduction by facilitating the influx of chloride ions into nerve cells through γ-aminobutyric acid (GABA) channels, leading to neuronal hyperpolarization. Ketamine functions as an analgesic by antagonizing the excitatory neurotransmitter glutamate, thereby preventing its binding to N-methyl-D-aspartate (NMDA) receptors. Local anesthetics, such as procaine and lidocaine, are widely recognized for their ability to interact with voltage-gated sodium channels, inducing channel inactivation and impeding the passage of sodium ions (Na+). This prevents the propagation of nerve impulses, achieving localized analgesia and inhibiting pain production (96). Moreover, local anesthetics can penetrate neurons via the lipid bilayer of neuronal membranes and through the TRPV-1 channel pathway, thereby exerting a nerve-blocking effect (96).

### 4.2 Effect of anesthesia on lactate

In a clinical trial assessing the comparative effects of ciprofol and propofol on anesthetized patients undergoing cardiac surgery, Yu et al. observed a reduction in lactate levels by an average of 0.1 mmol/L ten minutes post-anesthesia induction compared to the levels ten minutes pre-induction, for both pharmaceuticals (97). In contrast, within the context of postoperatively ventilated liver transplant patients administered a comparable dose of propofol, a comprehensive examination of propofol concentrations and lactate levels over a 14-hour period post-anesthesia revealed no discernible correlation between the anesthetic and lactate levels (98). Furthermore, Zou et al., in an experimental study involving the intraperitoneal injection of propofol in seven-day-old mice, noted an elevation in arterial blood lactate levels at the 6-hour and 12-hour marks post-injection when compared to the control cohort (99). These collective findings imply that propofol may possess the unique ability to diminish lactate concentrations in a manner independent of concentration gradients at therapeutic levels, yet may paradoxically stimulate lactate production at concentrations exceeding safety thresholds.

Dexmedetomidine (Dex), an α2-adrenoceptor agonist, is extensively utilized in the practice of general anesthesia. A wealth of clinical studies and animal experimental data indicate that Dex is efficacious in lowering serum lactate levels post-anesthesia, particularly in patients who have undergone myocardial ischemia-reperfusion injury and in corresponding rat models (100). Consistent with these observations, clinical trials employing Dex for anesthesia in various patient populations, including those undergoing coronary artery bypass grafting (101), living liver transplantation (102), critically ill patients facing gastrointestinal surgery (103), and non-diabetic individuals (104), have corroborated that Dex is associated with a reduction in lactic acid production across a spectrum of clinical scenarios. Furthermore, the implementation of a Dex-mediated long-term sedation protocol has been demonstrated to not only reduce lactate levels but also to significantly decrease mortality rates among patients with septic shock (105). Beyond its traditional anesthetic applications, Dex has been shown to inhibit glycolysis in macrophages, consequently dampening the pro-inflammatory response typically triggered by lipopolysaccharide (106). Collectively, these studies underscore the broad-ranging disease-ameliorating potential of Dex, extending beyond its role in anesthesia.

Ou et al. reported that a 6-hour treatment with 50 μM/L ketamine enhances glucose uptake in astrocytes via the ERK/GLUT3 signaling pathway, thereby stimulating lactate production (107). However, this finding contrasts with clinical study outcomes, which indicate that ketamine correlates with a decrease in lactate levels (108). This inconsistency may stem from differences in the sites of lactate detection.

Conner et al. confirmed in a porcine model that lidocaine alleviates severe ischemia-reperfusion injury, marked by a notable enhancement in lactate clearance (109). Ahuja et al. further explored the impact of lidocaine on serum lactate levels following anesthesia in patients undergoing intestinal surgery. Their study revealed that patients administered intraoperative lidocaine exhibited reduced postoperative lactate and LDH levels compared to the saline group, thereby improving postoperative outcomes (110). In a divergent finding, Pustetto et al.’s clinical trial indicated that lidocaine does not significantly affect blood lactate levels in patients undergoing major abdominal surgery (111), suggesting that the influence of lidocaine may vary depending on the disease model.

Levobupivacaine and ropivacaine new types of local anesthetic, which has the advantages of fast onset of action, long duration of action, and high safety, etc. Li et al. found that levobupivacaine could inhibit the proliferation and migration of colorectal adenocarcinoma cells, but had no anticancer effect on melanoma cells (112). In addition, levobupivacaine can inhibit the proliferation of breast cancer cells and promote the apoptosis of cancer cells *in vitro* (113). Levobupivacaine inhibited the growth of gastric cancer cells through the miR-489-3p/SLC7A11 axis, leading to ferroptosis of cancer cells (114)while in non-small cell lung cancer cells, levobupivacaine up-regulated p53, inhibited the progression of NSCLC and induced ferroptosis (115). Unfortunately, no study has yet explored the effects of levobupivacaine on lactate metabolism in cancer models. (For an overview of how anesthetics regulate lactate, refer to **Table 2**).

**Table 2:** The role of anesthetics in regulation of lactate.

Anesthetic	Disease	Lactate change	Ref.
Propofol	Patients undergoing cardiac surgery	↓	(97)
	Patients with liver transplant	irrelevant	(98)
	Mice at seven days of age	↑	(99)
Dexmedetomidine	Patients and rat with myocardial ischemia-reperfusion injury	↓	(100)
	Patients undergoing coronary artery bypass grafting	↓	(101)
	Patients with living liver transplantation	↓	(102)
	Critically ill patients undergoing gastrointestinal surgery	↓	(103)
	Non-diabetic patients	↓	(104)
	Patients with septic shock	↓	(105)
	LPS-induced proinflammatory responses in macrophages	↓	(106)
Ketamine	Depressive-like behaviors in mice	↑	(107)
	mechanically ventilated patients with SARS-CoV-2	↓	(108)
Lidocaine	Severe ischemia-reperfusion injury	↓	(109)
	Patients undergoing intestinal surgery	↓	(110)
	Patients undergoing major abdominal surgery	No change	(111)
Levobupivacaine			

↑ represents an upregulation of lactate levels and ↓ means an downregulation of lactate.

### 4.3 Potential impact of anesthesia on tumor progression

Propofol has been demonstrated to curb aerobic glycolysis in lung cancer cells by modulating the circ-ERBB2/miR-7-5p/FOXM1 axis, thereby suppressing cancer cell proliferation and invasion (116). However, a study by Hu et al. noted a discrepancy; while propofol inhibited lung cancer cell proliferation, invasion, and migration, it unexpectedly increased the levels of lactic acid and glucose in the culture medium of lung cancer cells (117). Potential reasons regarding this difference in lactate changes could be the result of different concentrations of isoproterenol administered as well as different drug treatment times (Gao et al. treated A59 cells for 48h using 5, 10 and 15 μg/ml isoproterenol and Hu treated A59 cells for 2h using 4 μg/mL isoproterenol). This finding contrasts with earlier research and intriguingly, they also observed that propofol lacked comparable anti-cancer efficacy against brain cancer (117). Yang et al. (118) and Dong et al. (119) have each concluded that propofol potently inhibits ovarian cancer cell proliferation, invasion, migration, and glycolysis through the regulation of circ_MUC16/miR-1182/S100B and circ-ZFR/miR-212-5p/SOD2, respectively. In contrast, Dexmedetomidine forestalls malignant progression of cells by inhibiting glycolysis and suppressing c-myc lactylation in glioblastoma cells (120). Peng et al. observed that lidocaine, while ineffective on neuropsychological cognitive function in patients undergoing supratentorial tumor surgery, successfully downregulated arterial and venous blood lactate levels (121). (For a detail of how anesthetics influence cancer progression and lactate levels, refer to **Table 3**).

**Table 3:** The role of anesthetics in cancer progression.

Anesthetic	Mechanism	Function	Lactate change	Ref.
Propofol	CircTADA2A/miR-455-3p/FOXM1	Inhibit lung cancer tumorigenesis and glycolysis	↓	(116)
	Down-regulate GLUT1, MPC1, HIF-1α, p-Akt and p-Erk1/2 expression and up-regulate PEDF	Suppress lung cancer cell viability, proliferation, migration and invasion of lung cancer cells	↑	(117)
	Unknown	Don’t have anti-cancer effects on brain cancer	Unknown	(117)
	Circ_MUC16/miR-1182/S100B	Inhibit ovarian cancer cell proliferation, migration, invasion and glycolytic metabolism	↓	(118)
	Circ-ZFR/miR-212-5p/SOD2	Inhibit ovarian cancer cell proliferation, migration, invasion and glycolytic metabolism	↓	(119)
Dexmedetomidine	Inhibit lactylation of c-myc	Suppress migration, invasion and glycolysis of glioblastoma cells	↓	(120)
Lidocaine	Unknown	Does not affect neuropsychological cognitive abilities of patients undergoing surgery for supratentorial tumors	↓	(121)

↑ represents an upregulation of lactate levels and ↓ means an downregulation of lactate.

When Propofol was used as an anesthetic in patients with breast cancer (122), non-small cell lung cancer (123) and bladder cancer (124) in clinical studies, there was no difference in long-term survival after cancer surgery when compared to inhalational anesthetics. However, in gynecological cancer surgery, propofol performed better than inhalational anesthetics in terms of overall survival, cancer-specific and recurrence-free survival (125). Killian suggests that although dexmedetomidine has been shown to alleviate the inflammatory stress response produced by surgery and thereby stimulate anticancer immunity in cancer therapy, there is a lack of prospective randomized trials investigating the effect of DEX administration on recurrence-free survival and overall survival, which greatly limits the use of dexmedetomidine in oncology anesthesia (126). In a clinical trial on the effect of lidocaine on cancer outcomes, we found that lidocaine intraoperative infusion was associated with prolonged overall and disease-free survival in patients with ovarian cancer (127). This may be related to the ability of lidocaine to inhibit the elevation of biomarkers (MPO and NE) associated with metastasis and recurrence (128), and to the fact that continuous intravenous infusion of lidocaine in the perioperative period significantly reduced the production of tumor metastasis biomarkers (NETs) (129). However, intraoperative lidocaine infusion did not improve overall survival or disease-free survival in pancreatic cancer patients undergoing pancreatectomy (130).

## 5 Discussion

Lactic acid, a metabolic byproduct of tumor cell glycolysis, accumulates in the tumor microenvironment (TME) as a hallmark of rapid tumor cell proliferation. Extensive research indicates that lactic acid functions as a promoter of tumor cell proliferation, invasion, and migration through a spectrum of direct and indirect effects (refer to **Figure 3**). Lactate has been demonstrated to exert its influence on amino acid metabolism (31) and fatty acid metabolism (34) through lactylation modifications, while also modulating glycolysis-related enzymes that regulate glucose and energy metabolism (32, 33). Despite numerous sequencing analyses implicating histone lysine lactylation (Kla) modifications in tumor cell metabolism (28, 29), there remains a dearth of correlative cellular experimental data, particularly concerning the molecular mechanisms through which lactate modulates metabolic pathways. In the realm of tumor immunity, lactate is recognized for its inhibitory effect on T cell proliferation (38). Moreover, lactic acid is shown to induce the differentiation of CD4+ cells into Treg cells (40) and the maturation of CD8+ cells into a memory phenotype (41). Additionally, lactic acid stimulates neutrophils to increase PD-L1 expression, thereby inhibiting T-cell-mediated tumor killing (42). Lactate also impacts macrophage function by disrupting polarization (47, 48), promoting the secretion of high-mobility group box protein (HMGB) to enhance tumor cell proliferation (44), and modulating the secretion of IL-1β by macrophages, which in turn influences the intricate interplay between tumor cell and macrophage recruitment (45). Collectively, these immunologically relevant findings underscore the multifaceted role of lactic acid in suppressing T cell tumoricidal functions, promoting macrophage polarization towards the M2 phenotype, and regulating the immunomodulatory interactions between macrophages and tumor cells, all of which are critical in the context of immune evasion. A new wave of technologies, such as poly-omics, provides a multidimensional perspective for studying tumor metabolism. At the transcriptome level, researchers have been able to identify a range of genes regulated by lactate, which are involved in a number of key processes such as cell cycle regulation, signal transduction, and metabolic reprogramming. At the metabolome level, researchers were able to accurately measure changes in the concentration of lactate and its related metabolites in tumor tissues, and found that lactate not only accumulates as a metabolite, but also participates in the feedback regulation of multiple metabolic pathways. In addition, the combination of metabolomics and genetics can reveal the link between metabolism and genetics and the potential mechanism of action, providing new perspectives for the discovery of novel drug targets and understanding of the underlying disease mechanisms (131). Whether lactylation modifications can also occur in RNA and DNA and thus promote tumor development or tumor therapy resistance is also a question to be explored in the future. Advances in mass spectrometry and high-throughput screening technologies may be a breakthrough in addressing these questions, which will be of great significance in defining the scope of lactylation and understanding tumor immunoregulation (132). In addition to the role of novel genomics technologies in studying the mechanisms by which lactate affects tumor progression and immune function, wearable devices integrated into hospital IT systems in clinical settings are also useful for monitoring lactate levels as a predictor of disease progression in patients. Lactate is now used clinically as a predictor of survival in critically ill patients, making continuous lactate monitoring essential (133, 134).

The association between lactate and pain is widely acknowledged, with the prevailing belief being that the accumulation of lactate is a precursor to pain. Empirical evidence has confirmed that lactate levels are indeed elevated in patients who report pain compared to asymptomatic individuals (79, 80). The presumed mechanism involves lactate accumulation activates acidic injury receptors, contributing to pain perception (81). However, emerging research challenges this notion, positing that lactate alone is insufficient to elicit pain and only induces fatigue and pain when co-injected with ATP and H+ intramuscularly (83). This challenges the established view of lactate as a significant pain inducer, indicating that the underlying mechanisms are more complex and remain to be fully elucidated. Recent studies have also highlighted the role of lactic acid in inducing pain through the stimulation of associated neurons. The inhibition of the lactate transporter protein MCT4 in the dorsal root ganglion (DRG) of sensory neurons has been shown to modulate neuronal excitation and induce pain (84). Moreover, the exchange of lactate between astrocytes and neurons has been identified as an effective strategy for mitigating chronic pain (85, 86). The synaptic accumulation of lactate is suggested to induce downstream neuronal death, contributing to central sensitization and the perpetuation of chronic pain (87). Furthermore, the exploration into the regulatory role of pain on lactate levels begins with the activation of the hypothalamic-pituitary-adrenal-thyroid-gonadal (HPATG) axis in response to pain. Progesterone’s involvement in lactate regulation is suggested; however, studies by Tomoka et al. (91) and Dragutinovic et al. (92) report contrasting findings regarding progesterone’s effect on lactate levels, potentially attributable to variations in exercise intensity and lactate testing duration between the studies. Further investigation is warranted to clarify these discrepancies. Marwali et al. demonstrated that exogenous thyroid supplementation does not influence lactate levels (94), a finding that appears to contradict the known role of thyroid hormones in enhancing glucose metabolism and, by extension, the expected impact on lactate metabolism.

Concluding our investigation, we assessed the impact of anesthetics on serum lactate levels among surgical patients. Our analysis revealed that the influence of Propofol and Ketamine on lactate concentrations is inconsistent across various studies, whereas Dexmedetomidine and Lidocaine exhibit a more predictable pattern, typically leading to a reduction in serum lactate levels. In the context of cancer patients, Propofol demonstrates a differential effect on lactate regulation, effectively preventing the progression of lung and ovarian cancers, yet showing no discernible anticancer activity against brain cancers. Dexmedetomidine not only lowers serum lactate levels but also curbs the invasiveness and migratory capabilities of glioblastoma cells. While Lidocaine has proven effective in diminishing lactate levels, its potential impact on cancer progression remains unexplored. For a comprehensive overview of how anesthetics modulate lactate levels and influence cancer outcomes, refer to **Tables 2** and **3**.

Overall, our review summarizes the role of lactate in tumor metabolism, tumor immunity and tumor progression, and finds that it may serve as a marker of malignant tumor progression. By exploring the interactions between pain and lactate, we found that lactate is not traditionally thought to be a single factor in triggering pain, but may induce pain by stimulating injury receptors as well as the nervous system. There are few studies that have investigated the mechanisms of lactate modulation by pain due to limitations in research thinking. Future investigations in this area should be increased, which will contribute to unravelling the blueprint of pain-lactate interactions and advancing the pleiotropic nature of pain treatment. While summarizing the studies on anesthetics on lactate and tumor progression, we found that Dexmedetomidine may be a promising drug for future anesthesia for oncology patients, not only to alleviate cancer pain but also to effectively reduce lactate levels to prevent cancer progression. Based on these findings, it is feasible to incorporate interventions targeting lactate metabolism, pain management, and anesthesia selection in order to develop a more effective integrated oncology treatment plan. For example, LDH and MCT inhibitors can reduce lactate production in tumor cells, thereby inhibiting tumor growth and invasion, suggesting that lactate metabolism-related enzyme inhibitors may have clinical applications to slow tumor progression. In addition, since some anesthetics may affect the energy metabolism of tumor cells, prompting the cells to shift to anaerobic glycolysis, thereby increasing lactate production, careful consideration is needed when selecting anesthetic drugs for tumor patients with abnormal lactate metabolism. Some anesthetic drugs, such as Dexmedetomidine, may have some antitumor effects and are able to reduce lactate levels to inhibit cancer progression; therefore, Dexmedetomidine may be a more appropriate choice for anesthesia and multimodal analgesia in tumor patients with abnormal lactate metabolism.

## References

[1] Peled E Assalia M Axelman L Norman D Nadir Y. PO-49 - Bone microinfarction and microcirculation thrombosis; is it a possible mechanism for bone pain among cancer patients? Thromb Res. (2016) 140 Suppl 1:S194–5. doi: 10.1016/S0049-3848(16)30182-7 27161735

[2] Haenen V Dams L Meeus M De Groef A. Altered somatosensory functioning and mechanism-based classification in breast cancer patients with persistent pain. Anat Rec (Hoboken). (2024) 307:273–84. doi: 10.1002/ar.v307.2 36398947

[3] Frasca M Martinez-Tapia C Jean C Chanteclair A Galvin A Bergua V. Serious health-related suffering impairs treatments and survival in older patients with cancer. J Pain Symp Manage. (2024) 68:506–515.e5. doi: 10.1016/j.jpainsymman.2024.08.002 39142494

[4] Scarborough BM Smith CB. Optimal pain management for patients with cancer in the modern era. CA Cancer J Clin. (2018) 68:182–96. doi: 10.3322/caac.21453 PMC5980731 29603142

[5] Fallon M Dierberger K Leng M Hall PS Allende S Sabar R. An international, open-label, randomised trial comparing a two-step approach versus the standard three-step approach of the WHO analgesic ladder in patients with cancer. Ann Oncol. (2022) 33:1296–303. doi: 10.1016/j.annonc.2022.08.083 36055465

[6] El-Boghdadly K Levy NA Fawcett WJ Knaggs RD Laycock H Baird E. Peri-operative pain management in adults: a multidisciplinary consensus statement from the Association of Anaesthetists and the British Pain Society. Anaesthesia. (2024) 79:1220–36. doi: 10.1111/anae.16391 39319373

[7] Doleman B Mathiesen O Sutton AJ Cooper NJ Lund JN Williams JP. Non-opioid analgesics for the prevention of chronic postsurgical pain: a systematic review and network meta-analysis. Br J Anaesth. (2023) 130:719–28. doi: 10.1016/j.bja.2023.02.041 PMC10251124 37059625

[8] Cai Q Liu G Huang L Guan Y Wei H Dou Z. The role of dexmedetomidine in tumor-progressive factors in the perioperative period and cancer recurrence: A narrative review. Drug Des Devel Ther. (2022) 16:2161–75. doi: 10.2147/DDDT.S358042 PMC9271281 35821701

[9] Lai HC Kuo YW Huang YH Chan SM Cheng KI Wu ZF. Pancreatic cancer and microenvironments: implications of anesthesia. Cancers (Basel). (2022) 14(11):2684. doi: 10.3390/cancers14112684 35681664 PMC9179559

[10] Brooks GA. Lactate as a fulcrum of metabolism. Redox Biol. (2020) 35:101454. doi: 10.1016/j.redox.2020.101454 32113910 PMC7284908

[11] Liberti MV Locasale JW. The warburg effect: how does it benefit cancer cells? Trends Biochem Sci. (2016) 41:211–8. doi: 10.1016/j.tibs.2015.12.001 PMC4783224 26778478

[12] Li X Yang Y Zhang B Lin X Fu X An Y. Lactate metabolism in human health and disease. Signal Transduct Target Ther. (2022) 7:305. doi: 10.1038/s41392-022-01151-3 36050306 PMC9434547

[13] Latham T Mackay L Sproul D Karim M Culley J Harrison DJ. Lactate, a product of glycolytic metabolism, inhibits histone deacetylase activity and promotes changes in gene expression. Nucleic Acids Res. (2012) 40:4794–803. doi: 10.1093/nar/gks066 PMC3367171 22323521

[14] Reuss AM Groos D Buchfelder M Savaskan N. The acidic brain-glycolytic switch in the microenvironment of Malignant glioma. Int J Mol Sci. (2021) 22(11):5518. doi: 10.3390/ijms22115518 34073734 PMC8197239

[15] Wang Y Li P Xu Y Feng L Fang Y Song G. Lactate metabolism and histone lactylation in the central nervous system disorders: impacts and molecular mechanisms. J Neuroinflamm. (2024) 21:308. doi: 10.1186/s12974-024-03303-4 PMC11605911 39609834

[16] Wang A Zou Y Liu S Zhang X Li T Zhang L. Comprehensive multiscale analysis of lactate metabolic dynamics *in vitro* and *in vivo* using highly responsive biosensors. Nat Protoc. (2024) 19:1311–47. doi: 10.1038/s41596-023-00948-y PMC12812421 38307980

[17] Warburg O. On the origin of cancer cells. Science. (1956) 123:309–14. doi: 10.1126/science.123.3191.309 13298683

[18] Medeiros HCD Lunt SY. The Warburg effect: Saturation of mitochondrial NADH shuttles triggers aerobic lactate fermentation. Mol Cell. (2022) 82:3119–21. doi: 10.1016/j.molcel.2022.08.004 PMC9888598 36055204

[19] Son J Lyssiotis CA Ying H Wang X Hua S Ligorio M. Glutamine supports pancreatic cancer growth through a KRAS-regulated metabolic pathway. Nature. (2013) 496:101–5. doi: 10.1038/nature12040 PMC3656466 23535601

[20] Chen Y Yang S Yu T Zeng T Wei L You Y. KDM4A promotes Malignant progression of breast cancer by down-regulating BMP9 inducing consequent enhancement of glutamine metabolism. Cancer Cell Int. (2024) 24:322. doi: 10.1186/s12935-024-03504-0 39300582 PMC11414211

[21] DeBerardinis RJ Mancuso A Daikhin E Nissim I Yudkoff M Wehrli S. Beyond aerobic glycolysis: transformed cells can engage in glutamine metabolism that exceeds the requirement for protein and nucleotide synthesis. Proc Natl Acad Sci U.S.A. (2007) 104:19345–50. doi: 10.1073/pnas.0709747104 PMC2148292 18032601

[22] Erb HHH Polishchuk N Stasyk O Kahya U Weigel MM Dubrovska A. Glutamine metabolism and prostate cancer. Cancers (Basel). (2024) 16(16):2871. doi: 10.3390/cancers16162871 39199642 PMC11352381

[23] Halestrap AP Wilson MC. The monocarboxylate transporter family–role and regulation. IUBMB Life. (2012) 64:109–19. doi: 10.1002/iub.v64.2 22162139

[24] Certo M Llibre A Lee W Mauro C. Understanding lactate sensing and signalling. Trends Endocrinol Metab. (2022) 33:722–35. doi: 10.1016/j.tem.2022.07.004 35999109

[25] Yu X Yang J Xu J Pan H Wang W Yu X. Histone lactylation: from tumor lactate metabolism to epigenetic regulation. Int J Biol Sci. (2024) 20:1833–54. doi: 10.7150/ijbs.91492 PMC10929197 38481814

[26] Yao S Chai H Tao T Zhang L Yang X Li X. Role of lactate and lactate metabolism in liver diseases (Review). Int J Mol Med. (2024) 54(1):59. doi: 10.3892/ijmm.2024.5383 38785162 PMC11188982

[27] Zhang D Tang Z Huang H Zhou G Cui C Weng Y. Metabolic regulation of gene expression by histone lactylation. Nature. (2019) 574:575–80. doi: 10.1038/s41586-019-1678-1 PMC6818755 31645732

[28] Hong H Chen X Wang H Gu X Yuan Y Zhang Z. Global profiling of protein lysine lactylation and potential target modified protein analysis in hepatocellular carcinoma. Proteomics. (2023) 23:2:e220043. doi: 10.1002/pmic.202200432 36625413

[29] Yang Z Yan C Ma J Peng P Ren X Cai S. Lactylome analysis suggests lactylation-dependent mechanisms of metabolic adaptation in hepatocellular carcinoma. Nat Metab. (2023) 5:61–79. doi: 10.1038/s42255-022-00710-w 36593272

[30] Sun J Li Y Chen R Xie Y Wei J Li B. Exploring the role of lactylation-related genes in osteosarcoma: A deep dive into prognostic significance and therapeutic potential. Environ Toxicol. (2024) 39:1001–17. doi: 10.1002/tox.24011 38009602

[31] Lu Y Zhu J Zhang Y Li W Xiong Y Fan Y. Lactylation-driven IGF2BP3-mediated serine metabolism reprogramming and RNA m6A-modification promotes lenvatinib resistance in HCC. Adv Sci (Weinh). (2024) 11(46):e2401399. doi: 10.1002/advs.202401399 39450426 PMC11633555

[32] Longhitano L Giallongo S Orlando L Broggi G Longo A Russo A. Lactate rewrites the metabolic reprogramming of uveal melanoma cells and induces quiescence phenotype. Int J Mol Sci. (2022) 24(1):24. doi: 10.3390/ijms24010024 36613471 PMC9820521

[33] Jiang J Huang D Jiang Y Hou J Tian M Li J. Lactate modulates cellular metabolism through histone lactylation-mediated gene expression in non-small cell lung cancer. Front Oncol. (2021) 11:647559. doi: 10.3389/fonc.2021.647559 34150616 PMC8208031

[34] Gao R Li Y Xu Z Zhang F Xu J Hu Y. Mitochondrial pyruvate carrier 1 regulates fatty acid synthase lactylation and mediates treatment of nonalcoholic fatty liver disease. Hepatology. (2023) 78:1800–15. doi: 10.1097/HEP.0000000000000279 36651176

[35] Li Z Li J Bai X Huang X Wang Q. Tumor microenvironment as a complex milieu driving cancer progression: a mini review. Clin Transl Oncol. (2024). doi: 10.1007/s12094-024-03697-w PMC12033186 39342061

[36] Shang S Wang MZ Xing Z He N Li S. Lactate regulators contribute to tumor microenvironment and predict prognosis in lung adenocarcinoma. Front Immunol. (2022) 13:1024925. doi: 10.3389/fimmu.2022.1024925 36505423 PMC9732022

[37] Yang H Zou X Yang S Zhang A Li N Ma Z. Identification of lactylation related model to predict prognostic, tumor infiltrating immunocytes and response of immunotherapy in gastric cancer. Front Immunol. (2023) 14:1149989. doi: 10.3389/fimmu.2023.1149989 36936929 PMC10020516

[38] Quinn WJ 3rd Jiao J TeSlaa T Stadanlick J Wang Z Wang L. Lactate limits T cell proliferation via the NAD(H) redox state. Cell Rep. (2020) 33:108500. doi: 10.1016/j.celrep.2020.108500 33326785 PMC7830708

[39] Yan M Yao J Lin Y Yan J Xie Y Fu Z. Tumor cell density dependent IL-8 secretion induces the fluctuation of tregs/CD8 + T cells infiltration in hepatocellular carcinoma: one prompt for the existence of density checkpoint. J Transl Med. (2023) 21:202. doi: 10.1186/s12967-023-04060-3 36932390 PMC10022186

[40] Tuomela K Levings MK. Acidity promotes the differentiation of immunosuppressive regulatory T cells. Eur J Immunol. (2023) 53:e2350511. doi: 10.1002/eji.202350511 37097063

[41] Wenes M Jaccard A Wyss T Maldonado-Pérez N Teoh ST Lepez A. The mitochondrial pyruvate carrier regulates memory T cell differentiation and antitumor function. Cell Metab. (2022) 34:731–746.e9. doi: 10.1016/j.cmet.2022.03.013 35452600 PMC9116152

[42] Deng H Kan A Lyu N He M Huang X Qiao S. Tumor-derived lactate inhibit the efficacy of lenvatinib through regulating PD-L1 expression on neutrophil in hepatocellular carcinoma. J Immunother Cancer. (2021) 9(6):e002305. doi: 10.1136/jitc-2020-002305 34168004 PMC8231064

[43] Shi Y Yasui M Hara-Chikuma M. AQP9 transports lactate in tumor-associated macrophages to stimulate an M2-like polarization that promotes colon cancer progression. Biochem Biophys Rep. (2022) 31:101317. doi: 10.1016/j.bbrep.2022.101317 35967760 PMC9372591

[44] Yan C Yang Z Chen P Yeh Y Sun C Xie T. GPR65 sensing tumor-derived lactate induces HMGB1 release from TAM via the cAMP/PKA/CREB pathway to promote glioma progression. J Exp Clin Cancer Res. (2024) 43:105. doi: 10.1186/s13046-024-03025-8 38576043 PMC10993467

[45] Xu C Xia Y Zhang BW Drokow EK Li HY Xu S. Macrophages facilitate tumor cell PD-L1 expression via an IL-1β-centered loop to attenuate immune checkpoint blockade. MedComm. (2020) 4:e242. doi: 10.1002/mco2.242 PMC10063777 37009412

[46] de-Brito NM Duncan-Moretti J da-Costa HC Saldanha-Gama R Paula-Neto HA Dorighello GG. Aerobic glycolysis is a metabolic requirement to maintain the M2-like polarization of tumor-associated macrophages. Biochim Biophys Acta Mol Cell Res. (2020) 1867:118604. doi: 10.1016/j.bbamcr.2019.118604 31760090

[47] Zhang A Xu Y Xu H Ren J Meng T Ni Y. Lactate-induced M2 polarization of tumor-associated macrophages promotes the invasion of pituitary adenoma by secreting CCL17. Theranostics. (2021) 11:3839–52. doi: 10.7150/thno.53749 PMC7914368 33664865

[48] Han S Bao X Zou Y Wang L Li Y Yang L. d-lactate modulates M2 tumor-associated macrophages and remodels immunosuppressive tumor microenvironment for hepatocellular carcinoma. Sci Adv. (2023) 9:eadg2697. doi: 10.1126/sciadv.adg2697 37467325 PMC10355835

[49] Romero M Miller K Gelsomini A Garcia D Li K Suresh D. Immunometabolic effects of lactate on humoral immunity in healthy individuals of different ages. Nat Commun. (2024) 15:7515. doi: 10.1038/s41467-024-51207-x 39209820 PMC11362567

[50] Feng X Ren J Zhang X Kong D Yin L Zhou Q. Lactate dehydrogenase A is implicated in the pathogenesis of B-cell lymphoma through regulation of the FER signaling pathway. Biofactors. (2024) 50:1024–38. doi: 10.1002/biof.v50.5 38516823

[51] Lopez E Karattil R Nannini F Weng-Kit-Cheung G Denzler L Galvez-Cancino F. Inhibition of lactate transport by MCT-1 blockade improves chimeric antigen receptor T-cell therapy against B-cell Malignancies. J Immunother Cancer. (2023) 11(6):e006287. doi: 10.1136/jitc-2022-006287 37399358 PMC10314680

[52] Brand A Singer K Koehl GE Kolitzus M Schoenhammer G Thiel A. LDHA-associated lactic acid production blunts tumor immunosurveillance by T and NK cells. Cell Metab. (2016) 24:657–71. doi: 10.1016/j.cmet.2016.08.011 27641098

[53] Ge W Meng L Cao S Hou C Zhu X Huang D. The SIX1/LDHA axis promotes lactate accumulation and leads to NK cell dysfunction in pancreatic cancer. J Immunol Res. (2023) 2023:6891636. doi: 10.1155/2023/6891636 36937004 PMC10022590

[54] Fu S He K Tian C Sun H Zhu C Bai S. Impaired lipid biosynthesis hinders anti-tumor efficacy of intratumoral iNKT cells. Nat Commun. (2020) 11:438. doi: 10.1038/s41467-020-14332-x 31974378 PMC6978340

[55] Li Z Wang Q Huang X Yang M Zhou S Li Z. Lactate in the tumor microenvironment: A rising star for targeted tumor therapy. Front Nutr. (2023) 10:1113739. doi: 10.3389/fnut.2023.1113739 36875841 PMC9978120

[56] Gu X Zhu Y Su J Wang S Su X Ding X. Lactate-induced activation of tumor-associated fibroblasts and IL-8-mediated macrophage recruitment promote lung cancer progression. Redox Biol. (2024) 74:103209. doi: 10.1016/j.redox.2024.103209 38861833 PMC11215341

[57] Apicella M Giannoni E Fiore S Ferrari KJ Fernández-Pérez D Isella C. Increased lactate secretion by cancer cells sustains non-cell-autonomous adaptive resistance to MET and EGFR targeted therapies. Cell Metab. (2018) 28:848–865.e6. doi: 10.1016/j.cmet.2018.08.006 30174307

[58] Chen J Huang Z Chen Y Tian H Chai P Shen Y. Lactate and lactylation in cancer. Signal Transduct Target Ther. (2025) 10:38. doi: 10.1038/s41392-024-02082-x 39934144 PMC11814237

[59] Yu H Li J Peng S Liu Q Chen D He Z. Tumor microenvironment: Nurturing cancer cells for immunoevasion and druggable vulnerabilities for cancer immunotherapy. Cancer Lett. (2024) 611:217385. doi: 10.1016/j.canlet.2024.217385 39645024

[60] Hanauer S Liedert B Balser S Brockstedt E Moschetti V Schreiber S. Safety and efficacy of BI 695501 versus adalimumab reference product in patients with advanced Crohn's disease (VOLTAIRE-CD): a multicentre, randomised, double-blind, phase 3 trial. Lancet Gastroenterol Hepatol. (2021) 6(10):816–25. doi: 10.1016/S2468-1253(21)00252-1 34388360

[61] Kefayat A Bahrami M Karami M Rostami S Ghahremani F. Veillonella parvula as an anaerobic lactate-fermenting bacterium for inhibition of tumor growth and metastasis through tumor-specific colonization and decrease of tumor’s lactate level. Sci Rep. (2024) 14:21008. doi: 10.1038/s41598-024-71140-9 39251652 PMC11385575

[62] Keller CR Martinez SR Keltz A Chen M Li W. Lactate oxidase disrupts lactate-activated RAS and PI3K oncogenic signaling. Cancers (Basel). (2024) 16(16):2817. doi: 10.3390/cancers16162817 39199589 PMC11353192

[63] Liu Y Suhail Y Novin A Afzal J Pant A. and Kshitiz, Lactate in breast cancer cells is associated with evasion of hypoxia-induced cell cycle arrest and adverse patient outcome. Hum Cell. (2024) 37:768–81. doi: 10.1007/s13577-024-01046-1 PMC11256967 38478356

[64] Piasentin N Milotti E Chignola R. The control of acidity in tumor cells: a biophysical model. Sci Rep. (2020) 10:13613. doi: 10.1038/s41598-020-70396-1 32788634 PMC7423962

[65] Meng S Sørensen EE Ponniah M Thorlacius-Ussing J Crouigneau R Larsen T. MCT4 and CD147 colocalize with MMP14 in invadopodia and support matrix degradation and invasion by breast cancer cells. J Cell Sci. (2024) 137(8):jcs261608. doi: 10.1242/jcs.261608 38661040 PMC11112124

[66] Ishihara S Hata K Hirose K Okui T Toyosawa S Uzawa N. The lactate sensor GPR81 regulates glycolysis and tumor growth of breast cancer. Sci Rep. (2022) 12:6261. doi: 10.1038/s41598-022-10143-w 35428832 PMC9012857

[67] Hou X Ouyang J Tang L Wu P Deng X Yan Q. KCNK1 promotes proliferation and metastasis of breast cancer cells by activating lactate dehydrogenase A (LDHA) and up-regulating H3K18 lactylation. PloS Biol. (2024) 22:e3002666. doi: 10.1371/journal.pbio.3002666 38905316 PMC11192366

[68] Chen L Xing X Zhu Y Chen Y Pei H Song Q. Palmitoylation alters LDHA activity and pancreatic cancer response to chemotherapy. Cancer Lett. (2024) 587:216696. doi: 10.1016/j.canlet.2024.216696 38331089

[69] Khajah MA Khushaish S Luqmani YA. The effect of lactate dehydrogenase inhibitors on proliferation, motility and invasion of breast cancer cells *in vitro* highlights a new role for lactate. Mol Med Rep. (2024) 29(1):12. doi: 10.3892/mmr.2023.13135 37997856 PMC10704548

[70] Bandopadhyay S Kamal IM Padmanaban E Ghosh DD Chakrabarti S Roy SS. Oncogene-mediated nuclear accumulation of lactate promotes epigenetic alterations to induce cancer cell proliferation. J Cell Biochem. (2023) 124:495–519. doi: 10.1002/jcb.v124.4 36999756

[71] Ma X Chen J Huang B Fu S Qu S Yu R. ErbB2-upregulated HK1 and HK2 promote breast cancer cell proliferation, migration and invasion. Med Oncol. (2023) 40:154. doi: 10.1007/s12032-023-02008-7 37079118

[72] Shao M Zhang J Zhang J Shi H Zhang Y Ji R. SALL4 promotes gastric cancer progression via hexokinase II mediated glycolysis. Cancer Cell Int. (2020) 20:188. doi: 10.1186/s12935-020-01275-y 32489324 PMC7247129

[73] Cai J Yi M Tan Y Li X Li G Zeng Z. Natural product triptolide induces GSDME-mediated pyroptosis in head and neck cancer through suppressing mitochondrial hexokinase-II. J Exp Clin Cancer Res. (2021) 40:190. doi: 10.1186/s13046-021-01995-7 34108030 PMC8188724

[74] Wu X Zhou Z Xu S Liao C Chen X Li B. Extracellular vesicle packaged LMP1-activated fibroblasts promote tumor progression via autophagy and stroma-tumor metabolism coupling. Cancer Lett. (2020) 478:93–106. doi: 10.1016/j.canlet.2020.03.004 32160975

[75] Xu J Feng X Yin N Wang L Xie Y Gao Y. Exosomes from cisplatin-induced dormant cancer cells facilitate the formation of premetastatic niche in bone marrow through activating glycolysis of BMSCs. Front Oncol. (2022) 12:922465. doi: 10.3389/fonc.2022.922465 36568212 PMC9786109

[76] Basbaum AI Bautista DM Scherrer G Julius D. Cellular and molecular mechanisms of pain. Cell. (2009) 139:267–84. doi: 10.1016/j.cell.2009.09.028 PMC2852643 19837031

[77] Santoni A Mercadante S Arcuri E. Chronic cancer and non-cancer pain and opioid-induced hyperalgesia share common mechanisms: neuroinflammation and central sensitization. Minerva Anestesiol. (2021) 87:210–22. doi: 10.23736/S0375-9393.20.14822-3 33300326

[78] Ji RR Xu ZZ Gao YJ. Emerging targets in neuroinflammation-driven chronic pain. Nat Rev Drug Discovery. (2014) 13:533–48. doi: 10.1038/nrd4334 PMC4228377 24948120

[79] Gerdle B Larsson B Forsberg F Ghafouri N Karlsson L Stensson N. Chronic widespread pain: increased glutamate and lactate concentrations in the trapezius muscle and plasma. Clin J Pain. (2014) 30:409–20. doi: 10.1097/AJP.0b013e31829e9d2a 23887335

[80] Boguszewicz Ł Heyda A Ciszek M Bieleń A Skorupa A Mrochem-Kwarciak J. Metabolite biomarkers of prolonged and intensified pain and distress in head and neck cancer patients undergoing radio- or chemoradiotherapy by means of NMR-based metabolomics-A preliminary study. Metabolites. (2024) 14:60. doi: 10.3390/metabo14010060 PMC10819132 38248863

[81] Kim TJ Freml L Park SS Brennan TJ. Lactate concentrations in incisions indicate ischemic-like conditions may contribute to postoperative pain. J Pain. (2007) 8:59–66. doi: 10.1016/j.jpain.2006.06.003 16949881

[82] Sluka KA Winter OC Wemmie JA. Acid-sensing ion channels: A new target for pain and CNS diseases. Curr Opin Drug Discovery Devel. (2009) 12:693–704. doi: 10.1016/j.jpain.2006.06.003 PMC3494879 19736627

[83] Pollak KA Swenson JD Vanhaitsma TA Hughen RW Jo D White AT. Exogenously applied muscle metabolites synergistically evoke sensations of muscle fatigue and pain in human subjects. Exp Physiol. (2014) 99:368–80. doi: 10.1113/expphysiol.2013.075812 PMC3946674 24142455

[84] Hasegawa K Okui T Shimo T Ibaragi S Kawai H Ryumon S. Lactate transporter monocarboxylate transporter 4 induces bone pain in head and neck squamous cell carcinoma. Int J Mol Sci. (2018) 19(2):368–80. doi: 10.3390/ijms19113317 PMC6274991 30366393

[85] Wang Y Peng Y Zhang C Zhou X. Astrocyte-neuron lactate transport in the ACC contributes to the occurrence of long-lasting inflammatory pain in male mice. Neurosci Lett. (2021) 764:136205. doi: 10.1016/j.neulet.2021.136205 34478818

[86] Hu Y Zou H Zhong Z Li Q Zeng Q Ouyang Q. The role of astrocyte-neuron lactate shuttle in neuropathic orofacial pain. J Oral Rehabil. (2024) 764:136205. doi: 10.1111/joor.v51.12 39209792

[87] Kong E Li Y Deng M Hua T Yang M Li J. Glycometabolism reprogramming of glial cells in central nervous system: novel target for neuropathic pain. Front Immunol. (2022) 13:861290. doi: 10.3389/fimmu.2022.861290 35669777 PMC9163495

[88] Tennant F. The physiologic effects of pain on the endocrine system. Pain Ther. (2013) 2:75–86. doi: 10.1007/s40122-013-0015-x 25135146 PMC4107914

[89] Isehunwa GO Oladun OT Akpan JE Alada ARA. Effect of Cortisol on Plasma Lactate Levels following Cortisolinduced Hyperglycaemia in Common African Toad, Bufo regularis. Niger J Physiol Sci. (2017) 32:21–5. doi: 10.1007/s40122-013-0015-x 29134973

[90] Grande G De Toni L Garolla A Milardi D Ferlin A. Plasma metabolomics in male primary and functional hypogonadism. Front Endocrinol (Lausanne). (2023) 14:1165741. doi: 10.3389/fendo.2023.1165741 37334300 PMC10273261

[91] Matsuda T Takahashi H Nakamura M Ogata H Kanno M Ishikawa A. Influence of the menstrual cycle on muscle glycogen repletion after exhaustive exercise in eumenorrheic women. J Strength Cond Res. (2023) 37:e273–9. doi: 10.1519/JSC.0000000000004306 35836304

[92] Dragutinovic B Moser F Notbohm HL Ihalainen JK Bloch W Schumann M. Influence of menstrual cycle and oral contraceptive phases on strength performance, neuromuscular fatigue, and perceived exertion. J Appl Physiol. (2024) 137:919–33. doi: 10.1152/japplphysiol.00198.2024 39052822

[93] Zamer BA Cui ZG Eladl MA Hamad M Muhammad JS. Estrogen treatment in combination with pyruvate kinase M2 inhibition precipitate significant cumulative antitumor effects in colorectal cancer. J Biochem Mol Toxicol. (2024) 38:e23799. doi: 10.1002/jbt.23799 39132768

[94] Marwali EM Caesa P Rayhan M Roebiono PS Fakhri D Haas NA. The effect of oral triiodothyronine supplementation on lactate and pyruvate after paediatric cardiac surgery. Cardiol Young. (2021) 31:205–11. doi: 10.1017/S1047951120003698 33168128

[95] Vats A Marbaniang MJ. The principles and conduct of anaesthesia. Surg (Oxford). (2022) 40:361–9. doi: 10.1016/j.mpsur.2022.04.002

[96] Jeske AH. Local anesthetics. In: Jeske AH, editor. Contemporary dental pharmacology: evidence-based considerations. Springer International Publishing, Cham (2024). p. 9–23.

[97] Yu L Liu X Zhao X Shan X Bischof E Lu HH. Ciprofol versus propofol for anesthesia induction in cardiac surgery: a randomized double-blind controlled clinical trial. BMC Anesthesiol. (2024) 24:412. doi: 10.1186/s12871-024-02795-0 39533186 PMC11556191

[98] Jaju R Prakash K Arora MK. Low-dose propofol as a solo agent for sedation in postoperative ventilated liver transplant recipients: A preliminary observational study. J Anaesthesiol Clin Pharmacol. (2023) 39:84–7. doi: 10.4103/joacp.joacp_169_21 PMC10220205 37250270

[99] Zou L Ning M Wang W Zheng Y Ma L Lv J. Naringenin prevents propofol induced neurodegeneration in neonatal mice brain and long-term neurocognitive impacts on adults. Drug Des Devel Ther. (2020) 14:5469–82. doi: 10.2147/DDDT.S280443 PMC7735719 33328725

[100] She H Hu Y Zhao G Du Y Wu Y Chen W. Dexmedetomidine ameliorates myocardial ischemia-reperfusion injury by inhibiting MDH2 lactylation via regulating metabolic reprogramming. Adv Sci (Weinh). (2024) 14:e2409499. doi: 10.1002/advs.202409499 PMC11672254 39467114

[101] Zhang C Zhang Y Liu D Mei M Song N Zhuang Q. Dexmedetomidine mitigates acute kidney injury after coronary artery bypass grafting: a prospective clinical trial. Rev Esp Cardiol (Engl Ed). (2024) 77:645–55. doi: 10.1016/j.recesp.2024.02.004 38423177

[102] Kwon HM Kang SJ Han SB Kim JH Kim SH Jun IG. Effect of dexmedetomidine on the incidence of postoperative acute kidney injury in living donor liver transplantation recipients: a randomized controlled trial. Int J Surg. (2024) 110:4161–9. doi: 10.1097/JS9.0000000000001331 PMC11254204 38537086

[103] Qi YP Ma WJ Cao YY Chen Q Xu QC Xiao S. Effect of dexmedetomidine on intestinal barrier in patients undergoing gastrointestinal surgery-A single-center randomized clinical trial. J Surg Res. (2022) 277:181–8. doi: 10.1016/j.jss.2022.03.031 35500513

[104] Zhou W Wang J Yang D Tian S Tan C Yang Y. Effects of dexmedetomidine on glucose-related hormones and lactate in non-diabetic patients under general anesthesia: a randomized controlled trial. Minerva Anestesiol. (2022) 88:8–15. doi: 10.23736/S0375-9393.21.15734-7 34709010

[105] Li L Shi X Xiong M Kong K Chen Z Zhou S. Dexmedetomidine only regimen for long-term sedation is associated with reduced vasopressor requirements in septic shock patients: A retrospective cohort study from MIMIC-IV database. Front Med (Lausanne). (2023) 10:1107251. doi: 10.3389/fmed.2023.1107251 36923011 PMC10010261

[106] Meng Q Guo P Jiang Z Bo L Bian J. Dexmedetomidine inhibits LPS-induced proinflammatory responses via suppressing HIF1α-dependent glycolysis in macrophages. Aging (Albany NY). (2020) 12:9534–48. doi: 10.18632/aging.103226 PMC7288940 32433037

[107] Ouyang X Wang Z Luo M Wang M Liu X Chen J. Ketamine ameliorates depressive-like behaviors in mice through increasing glucose uptake regulated by the ERK/GLUT3 signaling pathway. Sci Rep. (2021) 11:18181. doi: 10.1038/s41598-021-97758-7 34518608 PMC8437933

[108] Wyler D Torjman MC Leong R Baram M Denk W Long SC. Observational study of the effect of ketamine infusions on sedation depth, inflammation, and clinical outcomes in mechanically ventilated patients with SARS-CoV-2. Anaesth Intensive Care. (2024) 52:105–12. doi: 10.1177/0310057X231201184 38006606

[109] Conner J Lammers D Holtestaul T Jones I Kuckelman J Letson H. Combatting ischemia reperfusion injury from resuscitative endovascular balloon occlusion of the aorta using adenosine, lidocaine and magnesium: A pilot study. J Trauma Acute Care Surg. (2021) 91:995–1001. doi: 10.1097/TA.0000000000003388 34446655

[110] Ahuja V Singh K Thapa D Mitra S Attri AK Kaur J. Effect of lignocaine on postoperative serum lactate dehydrogenase and lactate levels in patients undergoing bowel surgery: A randomised controlled trial. Indian J Anaesth. (2024) 68:293–7. doi: 10.4103/ija.ija_948_23 PMC10926344 38476548

[111] Pustetto M Goldsztejn N Touihri K Engelman E Ickx B Van Obbergh L. Intravenous lidocaine to prevent endothelial dysfunction after major abdominal surgery: a randomized controlled pilot trial. BMC Anesthesiol. (2020) 20:155. doi: 10.1186/s12871-020-01075-x 32576151 PMC7310453

[112] Li T Chen L Zhao H Wu L Masters J Han C. Both Bupivacaine and Levobupivacaine inhibit colon cancer cell growth but not melanoma cells *in vitro* . J Anesth. (2019) 33:17–25. doi: 10.1007/s00540-018-2577-6 30426213 PMC6373235

[113] Kwakye AK Kampo S Lv J Ramzan MN Richard SA Falagán AA. Levobupivacaine inhibits proliferation and promotes apoptosis of breast cancer cells by suppressing the PI3K/Akt/mTOR signalling pathway. BMC Res Notes. (2020) 13:386. doi: 10.1186/s13104-020-05191-2 32807213 PMC7430121

[114] Mao SH Zhu CH Nie Y Yu J Wang L. Levobupivacaine induces ferroptosis by miR-489-3p/SLC7A11 signaling in gastric cancer. Front Pharmacol. (2021) 12:681338. doi: 10.3389/fphar.2021.681338 34177591 PMC8220201

[115] Meng M Huang M Liu C Wang J Ren W Cui S. Local anesthetic levobupivacaine induces ferroptosis and inhibits progression by up-regulating p53 in non-small cell lung cancer. Aging (Albany NY). (2021) 13:681338. doi: 10.18632/aging.203138 34175840

[116] Gao J Ding C Zhou J Wu G Han Z Li J. Propofol suppresses lung cancer tumorigenesis by modulating the circ-ERBB2/miR-7-5p/FOXM1 axis. Thorac Cancer. (2021) 12:824–34. doi: 10.1111/1759-7714.13856 PMC7952809 33506582

[117] Hu C Iwasaki M Liu Z Wang B Li X Lin H. Lung but not brain cancer cell Malignancy inhibited by commonly used anesthetic propofol during surgery: Implication of reducing cancer recurrence risk. J Adv Res. (2021) 31:1–12. doi: 10.1016/j.jare.2020.12.007 34194828 PMC8240101

[118] Yang H Guo Y Zhang Y Wang D Zhang G Hou J. Circ_MUC16 attenuates the effects of Propofol to promote the aggressive behaviors of ovarian cancer by mediating the miR-1182/S100B signaling pathway. BMC Anesthesiol. (2021) 21:297. doi: 10.1186/s12871-021-01517-0 34837947 PMC8626908

[119] Qu D Zou X Liu Z. Propofol modulates glycolysis reprogramming of ovarian tumor via restraining circular RNA-zinc finger RNA-binding protein/microRNA-212-5p/superoxide dismutase 2 axis. Bioengineered. (2022) 13:11881–92. doi: 10.1080/21655979.2022.2063649 PMC9275929 35543376

[120] Zhu J Zhang Y. Dexmedetomidine inhibits the migration, invasion, and glycolysis of glioblastoma cells by lactylation of c-myc. Neurol Res. (2024) 13(5):11881–92. doi: 10.1080/01616412.2024.2395069 39193894

[121] Peng Y Zhang W Zhou X Ji Y Kass IS Han R. Lidocaine did not reduce neuropsychological-cognitive decline in patients 6 months after supratentorial tumor surgery: A randomized, controlled trial. J Neurosurg Anesthesiol. (2016) 28:6–13. doi: 10.1097/ANA.0000000000000171 26083427

[122] Enlund M Berglund A Enlund A Lundberg J Wärnberg F Wang DX. Impact of general anaesthesia on breast cancer survival: a 5-year follow up of a pragmatic, randomised, controlled trial, the CAN-study, comparing propofol and sevoflurane. EClinicalMedicine. (2023) 60:102037. doi: 10.1016/j.eclinm.2023.102037 37333664 PMC10276257

[123] Gao Z Xu J Coburn M Ma D Wang K. Postoperative long-term outcomes and independent risk factors of non-small-cell lung cancer patients with propofol versus sevoflurane anesthesia: A retrospective cohort study. Front Pharmacol. (2022) 13:945868. doi: 10.3389/fphar.2022.945868 35935845 PMC9354745

[124] Enlund M Hållberg H Berglund A Sherif A Enlund A Bergkvist L. Long-term survival after volatile or propofol general anesthesia for bladder cancer surgery: A retrospective national registry cohort study. Anesthesiology. (2024) 140:1126–33. doi: 10.1097/ALN.0000000000004969 38466217

[125] Takeyama E Miyo M Matsumoto H Tatsumi K Amano E Hirao M. Long-term survival differences between sevoflurane and propofol use in general anesthesia for gynecologic cancer surgery. J Anesth. (2021) 35:495–504. doi: 10.1007/s00540-021-02941-9 34008073

[126] Carnet Le Provost K Kepp O Kroemer G Bezu L. Trial watch: dexmedetomidine in cancer therapy. Oncoimmunology. (2024) 13:2327143. doi: 10.1080/2162402X.2024.2327143 38481729 PMC10936656

[127] Zhang H Gu J Qu M Sun Z Huang Q Cata JP. Effects of intravenous infusion of lidocaine on short-term outcomes and survival in patients undergoing surgery for ovarian cancer: A retrospective propensity score matching study. Front Oncol. (2021) 11:689832. doi: 10.3389/fonc.2021.689832 35070949 PMC8770535

[128] Zhang W Liu J Li X Bai Z Sun Y Chen X. Lidocaine effects on neutrophil extracellular trapping and angiogenesis biomarkers in postoperative breast cancer patients with different anesthesia methods: a prospective, randomized trial. BMC Anesthesiol. (2024) 24:162. doi: 10.1186/s12871-024-02540-7 38678209 PMC11055234

[129] Ren B Cheng M Liu C Zheng H Zhang J Chen W. Perioperative lidocaine and dexmedetomidine intravenous infusion reduce the serum levels of NETs and biomarkers of tumor metastasis in lung cancer patients: A prospective, single-center, double-blinded, randomized clinical trial. Front Oncol. (2023) 13:1101449. doi: 10.3389/fonc.2023.1101449 36910600 PMC10003334

[130] Zhang H Qu M Guo K Wang Y Gu J Wu H. Intraoperative lidocaine infusion in patients undergoing pancreatectomy for pancreatic cancer: a mechanistic, multicentre randomised clinical trial. Br J Anaesth. (2022) 129:244–53. doi: 10.1016/j.bja.2022.03.031 35697547

[131] Bonanomi M Salmistraro N Fiscon G Conte F Paci P Bravatà V. Transcriptomics and metabolomics integration reveals redox-dependent metabolic rewiring in breast cancer cells. Cancers (Basel). (2021) 13(2):244–53. doi: 10.3390/cancers13205058 PMC8534001 34680207

[132] Hao ZN Tan XP Zhang Q Li J Xia R Ma Z. Lactate and lactylation: dual regulators of T-cell-mediated tumor immunity and immunotherapy. Biomolecules. (2024) 14(12):1646. doi: 10.3390/biom14121646 39766353 PMC11674224

[133] Yuzefpolskaya M Schwartz S Ladanyi A Abraham J Gale CP Grinstein J. The role of lactate metabolism in heart failure and cardiogenic shock: clinical insights and therapeutic implications. J Card Fail. (2025). doi: 10.1016/j.cardfail.2025.01.011 39890014

[134] Falter F Tisherman SA Perrino AC Kumar AB Bush S Nordström L. Serial lactate in clinical medicine - A narrative review. J Intensive Care Med. (2025), 8850666241303460. doi: 10.1177/08850666241303460 39925111 PMC12852492

